# Taste and odor metabolomics of *Schisandra chinensis* flesh and seeds: integration of sensory evaluation, electronic tongue, LC-QTOF-MS/MS, VirtualTaste, and HS-GC-IMS

**DOI:** 10.3389/fnut.2026.1881293

**Published:** 2026-08-05

**Authors:** Tian-Qi Zhang, Lichao Wang, Fang-Tong Liu, Gui-Zhong Xin, Hong-Juan Hou, Jinxiu Liang, Hui Zhang, Tian-Min Wang, De-Qiang Dou, Xiaowei Zhang, Da-Chuan Zhang, Cheng Qian, Long-Jiao Cong, Ting-Guo Kang, Gui-Rong Chen, Yue-Hua Chen, Hui-Peng Song

**Affiliations:** 1 Key Laboratory for Identification and Quality Evaluation of TCM of Liaoning Province, Liaoning University of Traditional Chinese Medicine, Dalian, China; 2 Department of Integration of Chinese and Western Medicine, School of Basic Medical Sciences, Peking University, Beijing, China; 3 State Key Laboratory of Natural Medicines, China Pharmaceutical University, Nanjing, China; 4 Institute of Genetics, International School of Medicine, Zhejiang University, Hangzhou, China; 5 Academy of Chinese Medical Sciences, Henan University of Chinese Medicine, Zhengzhou, China; 6 Department of Pathology, School of Basic Medical Sciences, Nanjing Medical University, Nanjing, China

**Keywords:** electronic tongue, HS-GC-IMS, LC-QTOF-MS, Schisandra chinensis, sensory evaluation

## Abstract

**Background:**

The fruit of *Schisandra chinensis* is a functional berry with a long application history and a complex flavor profile.

**Methods:**

To differentiate the flavor profiles of *Schisandra chinensis* flesh and seeds, an integrated analytical strategy combining sensory evaluation, electronic tongue, LC-QTOF-MS/MS, VirtualTaste, and HS-GC-IMS was developed.

**Results:**

Sensory evaluation and electronic tongue revealed that the flesh was characterized by sourness and lemon-like odor, while the seeds exhibited bitterness, pungency, and Sichuan pepper odor. LC-QTOF-MS/MS identified 57 potentially taste-active compounds. VirtualTaste prediction results revealed that citric acid and malic acid had high potential to produce sour taste, and these two acids might be major contributors to the sour sensation of the flesh. The contents of citric acid and malic acid in flesh were higher than those in seeds. Lignans exhibited high probabilities of bitter taste attribution in seeds. It was inferred that lignans such as schisantherin A, schisandrin A, and schisandrin B partially contributed to the bitter taste of seeds. These compounds accumulated abundantly in seeds and possessed certain bioactivities. The contents of schisantherin A, schisandrin A, and schisandrin B in seeds of the 18 sample batches ranged from 2.24–3.61, 2.91–4.06, and 3.23–4.33 mg/g, respectively. HS-GC-IMS analysis identified a total of 76 volatile compounds. Among them, *γ*-terpinene, 3-carene, *β*-pinene, and other components were more abundant in seeds than in flesh, and these substances were likely the key compounds responsible for pungency and the Sichuan pepper odor.

**Conclusion:**

This study clarified the differential distribution of flavor compounds in the fruit of Schisandra chinensis, providing key data for the separate utilization of the flesh and seeds.

## 1 Introduction

The fruit of *Schisandra chinensis*, known as the five-flavor fruit for its simultaneous sour, sweet, pungent, bitter, and salty flavors, possesses extensive biological functions (1–3). In fact, owing to its strong and pungent flavor, direct consumption of the fruit of *Schisandra chinensis* is generally poorly accepted. As a result, it is usually infused in water to reduce its intense taste. Although the sensory characteristics of the fruit of *Schisandra chinensis* have certain limitations, its health benefits such as liver protection, antioxidant activity, and sedative effects have been widely recognized (4). For example, Xu et al. (5) found that the extract of *Schisandra chinensis* relieved hepatocyte degeneration, thereby alleviating alcoholic liver injury. Yu et al. (6) demonstrated that certain compounds in *Schisandra chinensis* exhibited antioxidant effects. Chen et al. (7) discovered that the extract of *Schisandra chinensis* could regulate the overactivated HPA axis to exert anti-stress effects, thereby reversing stress-induced anxiety. Currently, the main active components of *Schisandra chinensis* have been identified as lignan compounds, such as schisandrin A and schisandrin B (8). Recent studies have demonstrated that these components possess cardiovascular-protective and anti-tumor effects. For instance, Luo et al. (9) discovered that schisandrin B could specifically target MyD88 in myocardial cells, thus alleviating inflammatory diabetic cardiomyopathy. Xu et al. (10) uncovered that schisandrin A was able to induce cell-cycle arrest and apoptosis by regulating the Wnt/ER stress signaling pathway, thereby effectively suppressing triple-negative breast cancer in preclinical models. However, the industrial exploitation of *Schisandra chinensis* fruits as functional foods still faces numerous limitations. This is mainly due to inadequate basic research regarding its sensory attributes. On the one hand, earlier studies mainly focused on bioactive ingredients, while few systematically explored the flavor material basis of *Schisandra chinensis* via tissue separation. On the other hand, the distribution patterns of flavor substances within different tissues (the flesh and seeds) of the fruit remain unclear. These limitations have substantially hindered the industrial food exploitation of the fruit of *Schisandra chinensis*. Firstly, the unclear chemical composition of flavor substances results in a lack of targeted flavor optimization, making it difficult to satisfy consumers’ taste demands. Secondly, the lack of knowledge about tissue-distribution characteristics has impeded the differential utilization of the flesh and seeds, thereby restricting the development of product diversity for the fruit of *Schisandra chinensis*. Therefore, uncovering the flavor substances and their distribution patterns in the fruit of *Schisandra chinensis* is of great research significance and can offer crucial scientific support for its food development.

The analysis of samples with complex flavors necessitates the combined application of multiple techniques. In recent years, common flavor research methods have included sensory evaluation (11, 12), electronic sensory technology (13, 14), liquid chromatography-mass spectrometry (15, 16), and headspace gas chromatography-ion mobility spectrometry (17, 18). Sensory evaluation employs human senses like smell and taste for the assessment of sample flavor profiles, which delivers the strengths of authenticity and superior sensitivity. For example, Predieri et al. (19) employed sensory evaluation to compare the flavor profiles of saffron from three Italian regions of Sardinia, central Tuscany, and southern Tuscany. The results revealed that samples from both Sardinia and southern Tuscany exhibited a more intense flavor. However, sensory evaluation is inherently limited by subjective bias and inter-individual variation. To overcome these limitations, electronic sensory systems were developed to quantify food flavors. For example, the electronic tongue, with its unique sensor array and pattern recognition system, was capable of rapidly and accurately identifying taste characteristics in samples. Huang et al. (20) employed an electronic tongue to analyze the flavor profiles of loquat fruit at different storage periods. They observed a gradual decline in flavor intensity with prolonged storage time. Zhao et al. (21) utilized an electronic tongue to analyze the taste characteristics of three distinct parts of wampee fruit. Their findings revealed differences in the taste profiles among these parts. In many instances, conducting merely a flavor evaluation of food is insufficient; a comprehensive analysis of its flavor composition is necessary. At present, the representative techniques for flavor component analysis are liquid chromatography-mass spectrometry (LC–MS) and headspace gas chromatography-ion mobility spectrometry (HS-GC-IMS). The LC-QTOF-MS/MS technique combined the high separation efficiency of liquid chromatography (LC) with the high resolution of quadrupole time-of-flight tandem mass spectrometry (QTOF-MS/MS), enabling the accurate determination of the chemical structures of taste components. For example, Molina-Calle et al. (22) employed LC-QTOF-MS/MS to analyze *Stevia rebaudiana*, leading to the preliminary identification of eighty-nine potential taste components. The LC-QTOF-MS/MS technique is capable of analyzing non-volatile components (23), whereas the analysis of volatile components requires the use of HS-GC-IMS (24). HS-GC-IMS combined the high separation capability of gas chromatography with the rapid analysis advantage of ion mobility spectrometry, enabling the qualitative and semi-quantitative analysis of volatile compounds. Wu et al. (25) identified seven common volatile organic compounds in the pericarp and leaves of *Zanthoxylum bungeanum* using HS-GC-IMS. Additionally, several other techniques are employed for the analysis of complex flavor samples. VirtualTaste is a computational platform that predicts the sweet, bitter, and sour tastes of chemical compounds using random forest algorithms as its core framework. Designed for early-stage virtual screening of food additives and drug candidates, the platform also provides precomputed taste profiles for approved pharmaceuticals and natural products (26). For instance, Sun et al. (27) combined VirtualTaste with HPLC-QTOF-MS to identify distinct bitter compounds in Gougu tea and Gonglao tea. However, its predictive performance is constrained by the quality of training datasets, the limited scope of the three basic taste types, and neglect of the concentration-related sensory impacts. It is evident that a single technique is insufficient for the comprehensive analysis of samples with complex flavors. Consequently, the combined application of multiple techniques becomes an inevitable trend in complex flavor analysis.

A research gap exists regarding the complex flavor profile of the fruit of *Schisandra chinensis*, as well as the unresolved tissue distribution patterns and material basis underlying flavor formation between flesh and seeds. To address this gap, this study constructed an integrated flavor analytical workflow integrating five complementary platforms: sensory evaluation, electronic tongue, LC-QTOF-MS/MS, VirtualTaste, and HS-GC-IMS. This specific combination was driven by the complementary advantages of each technique. Sensory evaluation enables comprehensive flavor profiling yet is hampered by individual subjective bias and imprecise quantification of flavor intensity, while the electronic tongue yields objective quantitative data for fundamental tastes (e.g., sourness, bitterness, saltiness, astringency, umami) but suffers from limited sensor coverage. Accordingly, the combined use of these approaches could facilitate broader analytical coverage and yield more reliable results for global flavor profiling (28). However, since neither approach can identify specific compounds, LC-QTOF-MS/MS was employed for structural characterization, and VirtualTaste was subsequently applied to predict the taste attributes of the identified substances (27). The resulting compound taste data, combined with relative content information, were used to explain flavor differences between the flesh and seeds. Nevertheless, given that LC-QTOF-MS/MS cannot detect volatile components, HS-GC-IMS was adopted for volatile identification, offering the advantages of rapid, high-sensitivity detection with minimal sample pretreatment (29). As such, this multi-technique strategy supports a comprehensive analysis covering both taste-active and volatile compounds, effectively overcoming the inherent limitations of single-technique approaches in complex sample analysis.

This study applied the established technical system to investigate the fruit of *Schisandra chinensis*, with two main objectives. The first was to characterize the distribution of taste- and odor-active compounds in the flesh and seeds, thereby revealing the core drivers of their flavor differences. The second was to analyze the composition of these compounds across both tissues, focusing on their types and content variations. Special attention was paid to the bitter-tasting lignans in the seeds, which exhibit a dual nature as both flavor substances and bioactive compounds. Ultimately, this research aimed to fill the existing gap in tissue-specific flavor distribution studies, providing essential data and a technical framework for flavor improvement and differentiated utilization of *Schisandra chinensis* flesh and seeds.

## 2 Materials and methods

### 2.1 Reagents and materials

Eighteen batches of *Schisandra chinensis* samples from Fushun (Liaoning province) were used. The fruit of *Schisandra chinensis* is a fleshy aggregate berry, with the seeds embedded in the succulent mesocarp. Each seed is reniform and has a smooth, yellowish-brown testa. The fruits were harvested in September 2024, and all materials belonged to the species *Schisandra chinensis*. The samples were sun-dried following standard operating procedures and subsequently stored in a refrigerator at 4 °C (30). The researchers wore food-grade gloves and manually separated the flesh from the seeds. The separated flesh and seeds were ground separately with a pulverizer and sieved through a 60-mesh sieve. The moisture content of the samples, determined by the toluene method, ranged from 12 to 15%, which met the national standard (31). The batch numbers of the 18 sample lots ranged from WZ-Y01 to WZ-Y18. Based on the Euclidean distances calculated from the lignan contents, batches 9 (WZ-Y09), 5 (WZ-Y05), and 4 (WZ-Y04) were selected as representative samples for sensory evaluation and HS-GC-IMS analysis. For the electronic tongue, LC-QTOF-MS/MS, and HPLC analyses, all 18 independent batches were tested individually. Schisandrin A, schisantherin A, schisandrol A, schisandrol B, schisandrin B, and schisanhenol (purity >98%) were from HerbPurify Co., Ltd. Schisantherin B, citric acid, protocatechuic acid, and malic acid (purity >98%) were from Push Bio-Technology Co., Ltd. HPLC-grade methanol and acetonitrile were purchased from Yongda Chemical Reagent Co., Ltd. and Merck, respectively. Purified water was provided by Wahaha Group.

### 2.2 Sample extraction

For LC-QTOF-MS/MS and electronic tongue analyses, two extraction systems were prepared, both following identical single-round procedures of ultrasonication, centrifugation, and filtration. Briefly, 0.25 g of pulverized flesh and seed samples were weighed into centrifuge tubes and extracted once with 18 mL of 80% methanol (solid–liquid ratio, 1:72) via ultrasonication for 30 min at 20 °C. Separately, 0.5 g of each sample powder was mixed with 100 mL of purified water (solid–liquid ratio, 1:200) and subjected to one round of the same ultrasonication for 30 min at 20 °C. Both mixtures were centrifuged at 12,000 rpm for 10 min, and the resulting supernatants were collected and filtered through a 0.22 μm microporous filter membrane to obtain the test solutions for LC-QTOF-MS/MS and electronic tongue analysis, respectively.

### 2.3 Sensory evaluation of *Schisandra chinensis* flesh and seeds

Seventy-six participants (56 females, 20 males) took part in the sensory evaluation after signing informed consent forms. Prior to the formal experiment, we conducted a small-scale preliminary survey involving 10 male and 10 female panelists. The results indicated that gender had no significant effect on sensory outcomes, which was consistent with our previously published findings (28). Accordingly, the gender ratio of formal assessors followed natural distribution without artificial screening. Participants aged 19 to 26 were recruited due to their sensitive and mature gustatory ability, which allowed precise description of the flavor characteristics of the fruit of *Schisandra chinensis*. All assessors were trained panelists with regular consumption experience of berry fruits including strawberries, blueberries and goji berries. Before formal testing, all panelists received standardized pre-experiment training in the sensory laboratory. The training consisted of theoretical lectures clarifying the definitions, sensory descriptors and scoring criteria of target flavors. The definitions of attribute descriptors were derived from *Sensory analysis—Vocabulary* (ISO 5492:2008, MOD). A series of practical discrimination exercises were also conducted. Specifically, Sichuan peppercorns were used as reference samples for training on recognizing the Sichuan pepper odor, while lemons served as references for practical identification of sourness and lemon-like odor.

Each assessor completed tests in an independent sensory booth with stable ambient temperature and neutral natural light that would not bias sensory judgment. Every participant was given 15 min and 30 intact *Schisandra chinensis* fruits for repeated odor and taste assessment to minimize random error from single sampling. Panelists rinsed their mouths thoroughly with purified water and took a 1-h break between sample sets to avoid taste and smell fatigue. All samples were anonymously labelled with unique three-digit random codes only on sample containers, with no original information such as producing regions or batches displayed. Quantitative sensory data were collected via standardized questionnaires, where each flavor intensity was scored on a 0–5 rating scale. From an ethical perspective, all participants signed written informed consent before sensory testing to confirm voluntary participation and grant permission for their sensory data to be used for research purposes. All personal identifiable information was securely stored and kept strictly confidential. The research team safeguarded participants’ legal rights and privacy, and all subjects were notified that they could withdraw from the trial at any time without penalty.

### 2.4 Conditions of electronic tongue

Taste analysis was performed by an SA-402B system (Intelligent Sensor Technology, Japan) with five lipid membrane sensors (sourness, bitterness, astringency, umami, saltiness) and two reference electrodes. A reference solution (artificial saliva) was used for calibration and cleaning. Sensors were equilibrated for 30 s before measurement. Each sample was tested in quadruplicate.

### 2.5 Conditions of LC-QTOF-MS/MS

Chemical analysis was performed using an Agilent 1290 HPLC system coupled with a 6550 Q-TOF MS. LC-QTOF-MS/MS was adopted for qualitative analysis, whereas HPLC was used for quantitative analysis. The parameters for qualitative analysis were as follows. Separation was performed on a phenyl-hexyl column (4.6 × 50 mm, 3.5 μm) with a mobile phase of 0.1% formic acid (A) and acetonitrile (B) at 0.5 mL/min. The gradient elution was performed with the following procedure: 0–6 min, 5–30% B; 6–40 min, 30–60% B; 40–49 min, 60–75% B; 49–52 min, 75–95% B; 52–55 min, 95–100% B; 55–60 min, 100–100% B. The column temperature was set at 30 °C. Analysis was performed using the Q-TOF-MS/MS system in both positive and negative ion modes. The operating parameters were configured as follows: gas temperature at 200 °C, gas flow rate at 11 L/min, sheath gas temperature at 350 °C, sheath gas flow rate at 8 L/min, nebulizer gas pressure at 35 psig, fragmentor voltage at 75 V, and m/z range from 100 to 3,000. The collision energy was set to 30 eV. Data were acquired in positive/negative ion modes using Agilent MassHunter. The quantitative analysis conditions were as follows. An AQ-C18 column (4.6 × 250 mm, 5 μm) was used. The target compounds were schisantherin A, schisandrin A and schisandrin B. A mixed standard stock solution containing these compounds was prepared. The concentrations of schisantherin A, schisandrin A, and schisandrin B in the stock solution were 0.75 mg/mL, 1.03 mg/mL, and 0.73 mg/mL, respectively. The stock solution of all three compounds was diluted to seven concentration levels. The standard curve was generated using linear least-squares regression in Microsoft Excel (version 2021). The mobile phase consisted of 0.1% phosphoric acid in water (A) and acetonitrile (B), with a flow rate of 1 mL/min. The gradient elution program was set as follows: 0–5 min, 20–30% B; 5–12 min, 30–50% B; 12–30 min, 50–60% B; 30–35 min, 60–65% B; 35–36 min, 65–80% B; 36–45 min, 80–80% B; 45–45.1 min, 80–100% B; 45.1–50 min, 100–100% B. The column temperature was set to 30 °C. The injection volume was 10 μL. The detection wavelength was 260 nm. Due to different analytical objectives and instrument properties, LC‑QTOF‑MS/MS allows qualitative identification without stringent chromatographic separation, whereas HPLC employs optimized conditions to ensure stable signals and accurate quantification.

### 2.6 Conditions of VirtualTaste prediction

Compound structures from PubChem^1^ were converted to ChemDoodle JSON format using Open Babel (version 3.1.1). Taste (bitterness, sourness) was predicted via the VirtualTaste model (version 2021)^2^. The VirtualTaste website was accessed from January 15, 2025 to February 28, 2025. All 57 compounds identified by LC-QTOF-MS/MS in this study were uploaded to the platform for taste prediction without prior screening. If the prediction result was labeled “Inactive,” it suggested that the compound was unlikely to exert the corresponding taste activity. Otherwise, a predicted value closer to 1 indicated a higher probability of possessing the corresponding taste attribute.

### 2.7 Conditions of HS-GC-IMS

Analysis was performed using a HS-GC-IMS system (FlavorSpec®, GAS Instrument, Dortmund, Germany) equipped with an MXT-5 capillary column (15 m × 0.53 mm, 1.0 μm; Restek, Inc., United States), a CTC-PAL 3 static headspace autosampler (CTC Analytics AG, Zwingen, Switzerland), and 20 mL headspace vials (Shandong Hanon Scientific Instrument Co., Ltd.). Data were processed using VOCal software (version 0.4.03, GAS, Dortmund, Germany). Each sample (1 g) was transferred to a 20 mL headspace vial and incubated at 80 °C for 20 min. Subsequently, a 500 μL aliquot was injected in splitless mode. The incubation agitation speed was set at 500 r/min, while the syringe and inlet temperatures were maintained at 85 °C and 80 °C, respectively. High-purity nitrogen (purity ≥99.999%) was used as the carrier gas. The flow rate of drift gas was 75 mL/min. The drift tube temperature was kept at the instrument default value of 45 °C, which is appropriate for the separation and identification of the majority of flavor compounds. The gas chromatography was operated with a programmed pressure gradient: initial flow rate of 2.0 mL/min held for 2 min; increased linearly to 10.0 mL/min over 8 min; then raised linearly to 100.0 mL/min over 10 min and maintained for 30 min. The total chromatographic run time was 50 min. The experiment was carried out in three independent biological replicates. Retention indices of volatile organic compounds (VOCs) were calculated using n-ketones as reference substances. Spectral profiles of volatile components were generated using VOCal data processing software for comparative analysis of VOCs among samples.

### 2.8 Data analysis

OPLS-DA was conducted using SIMCA 14.1. OPLS-DA model reliability was validated by a permutation test (*n* = 200). Variables with VIP > 1 were considered significant. Radar charts, heatmaps, bar graphs, and box plots were generated using Origin 2022 and GraphPad Prism 6.02.

## 3 Results

### 3.1 Combined application of five techniques in taste and odor metabolomics

**Figure 1:**
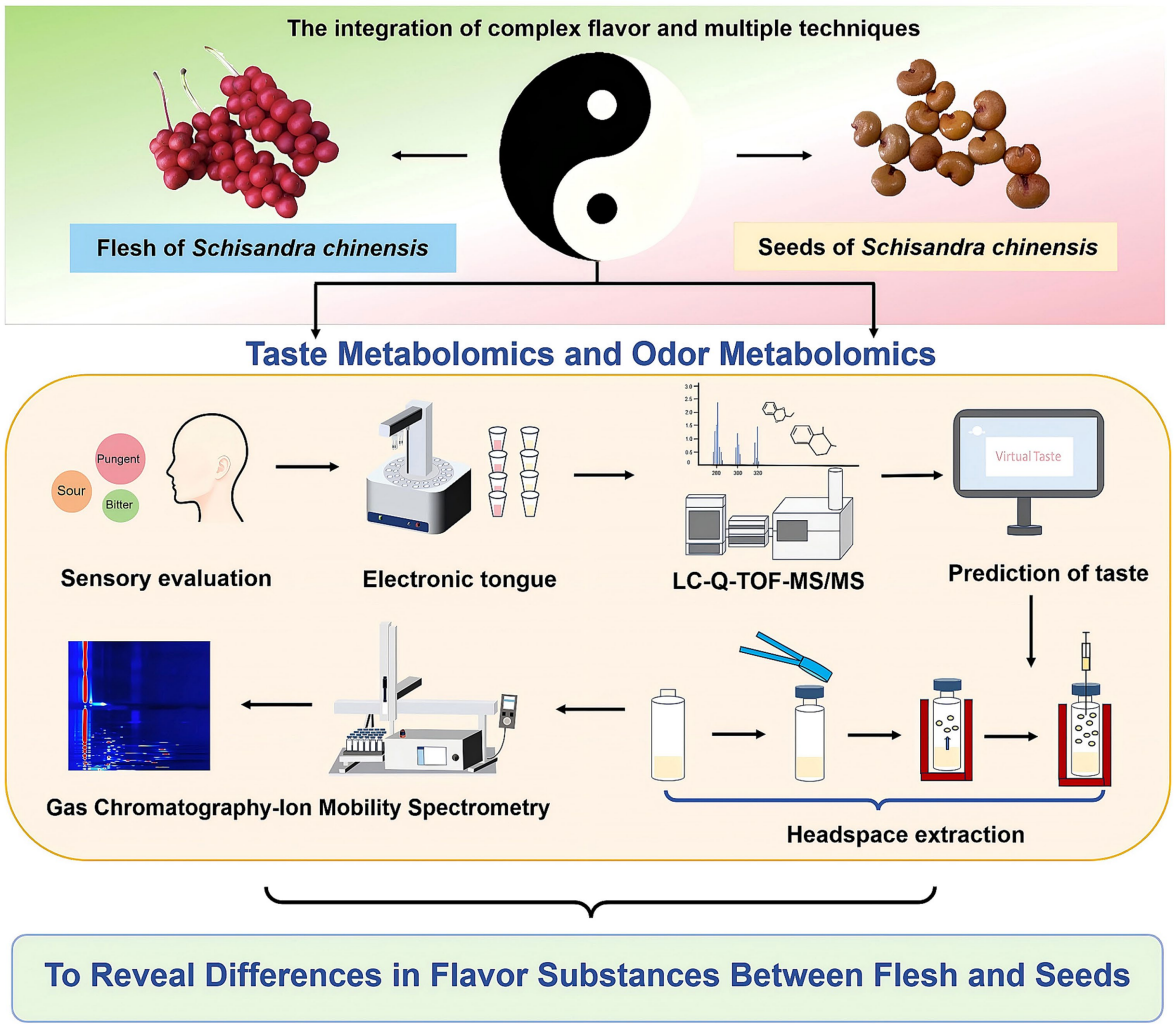
Integrated analytical workflow for taste and odor metabolomics to reveal differences in flavor substances between the flesh and the seeds of *Schisandra chinensis*.

To resolve the chemical composition and distribution patterns of complex flavor constituents in the fruits of *Schisandra chinensis*, this study proposed an integrated technical strategy (Figure 1). The strategy combined five analytical techniques for parallel detection. Sensory evaluation, electronic tongue, LC-QTOF-MS/MS, VirtualTaste, and HS-GC-IMS were integrated to characterize the flavor profiles of the fruit of *Schisandra chinensis* from taste and odor dimensions. Among these approaches, four complementary methods including sensory evaluation, electronic tongue, LC-QTOF-MS/MS, and VirtualTaste were applied for taste metabolomics analysis, which systematically uncovered the chemical basis of taste-related compounds in the flesh and seeds of *Schisandra chinensis*. Meanwhile, HS-GC-IMS was adopted for odor metabolomics testing to comprehensively illustrate the overall flavor components and their tissue distribution characteristics in the fruit of *Schisandra chinensis*. For taste characterization, sensory evaluation and electronic tongue were jointly used to profile the taste attributes of the flesh and seeds and identify the distribution patterns of taste-active compounds across different tissues. LC-QTOF-MS/MS was employed to identify chemical constituents in the flesh and seeds and compare their relative abundances. VirtualTaste was utilized to forecast the sour and bitter taste properties of all identified compounds. The synergistic application of the four techniques systematically screened taste-active substances in *Schisandra chinensis* and clarified their distribution patterns in distinct fruit tissues. For odor analysis, HS-GC-IMS was used for odor metabolomics assays to precisely separate and identify volatile aromatic compounds in flesh and seeds. The integration of these five analytical techniques yielded complementary metabolomic data covering taste and odor, which clarifies the compositional characteristics and tissue distribution of flavor compounds in the fruit of *Schisandra chinensis*.

### 3.2 Sensory evaluation

**Figure 2:**
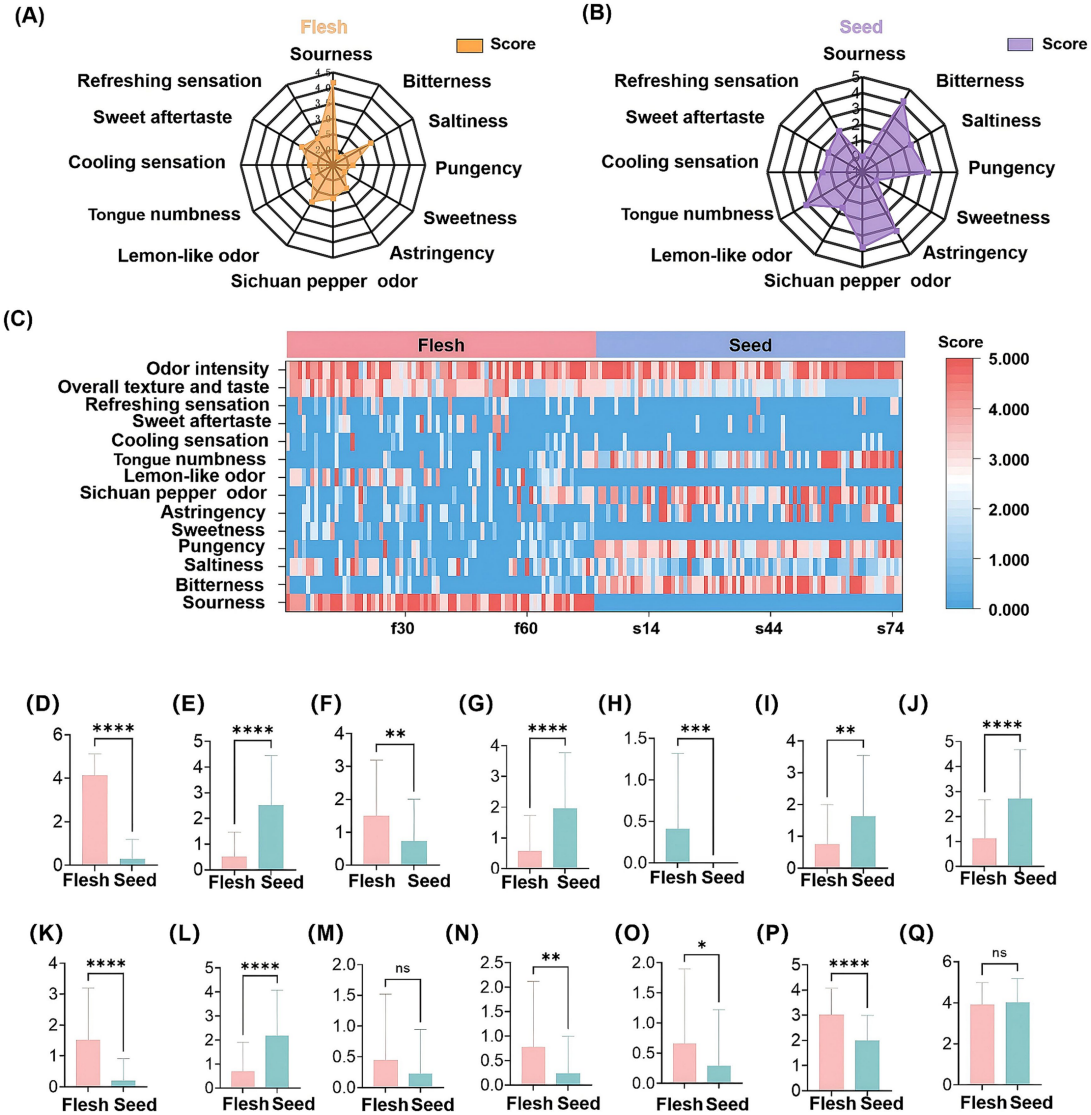
**(A)** Sensory evaluation radar chart of the flesh; **(B)** Sensory evaluation radar chart of the seeds; **(C)** Sensory evaluation heatmap of the flesh and seeds; Comparison of 14 sensory indicators including **(D)** sourness, **(E)** bitterness, **(F)** saltiness, **(G)** pungency, **(H)** sweetness, **(I)** astringency, **(J)** Sichuan pepper odor, **(K)** lemon-like odor, **(L)** tongue numbness, **(M)** cooling sensation, **(N)** sweet aftertaste, **(O)** refreshing sensation, **(P)** overall texture and taste, **(Q)** odor intensity between flesh and seeds. Independent *t*-tests were used (ns indicates no significant difference; *indicates 0.01 < *p* ≤ 0.05; **indicates 0.001 < *p* ≤ 0.01; ***indicates 0.0001 < *p* ≤ 0.001; ****indicates *p* < 0.0001).

To reveal the flavor characteristics and distribution patterns of the fruit of *Schisandra chinensis*, we analyzed the sensory evaluation data from 76 participants. The flavor characteristics of the flesh and the seeds are presented in Figures 2A, B, respectively. The sensory evaluation heatmap (Figure 2C) showed that the flesh scored higher in sourness and lemon-like odor than in other attributes, whereas the seeds exhibited the most pronounced intensities in bitterness, pungency, and Sichuan pepper odor. Saltiness was detected in both the flesh and the seeds, though their salty intensity remained relatively low. Only faint sweetness was perceived in flesh, and no sweetness was detected in seeds. Sichuan pepper odor, astringency, and tongue numbness were primarily concentrated in the seeds, while these characteristics were not prominent in the flesh. The flesh received high scores for its sweet aftertaste, while no significant differences were found between the two parts in terms of cooling sensation and refreshing sensation. The overall texture and taste evaluation showed that participants preferred the flesh. In terms of odor intensity, both the flesh and the seeds exhibited intense aromatic characteristics.

To differentiate the flavor characteristics of flesh and seeds, t-tests were applied to all sensory indices presented in the heatmap for comparison of each sensory attribute across the two tissues (Figures 2D–Q). Statistical results indicated extremely significant differences (*p* < 0.0001) between the flesh and seeds in sourness, bitterness, pungency, Sichuan pepper odor, lemon-like odor, tongue numbness, and overall texture and taste. Significant intergroup differences were observed for sweetness (0.0001 < *p* ≤ 0.001). Saltiness, astringency and sweet aftertaste had significant differences at 0.001 < *p* ≤ 0.01, while only marginally significant differences were found for refreshing sensation (0.01 < *p* ≤ 0.05). No statistically significant differences existed between the flesh and seeds in cooling sensation and odor intensity. The chemical substances responsible for the dominant flavor characteristics were further characterized in subsequent studies.

### 3.3 Electronic tongue analysis

**Figure 3:**
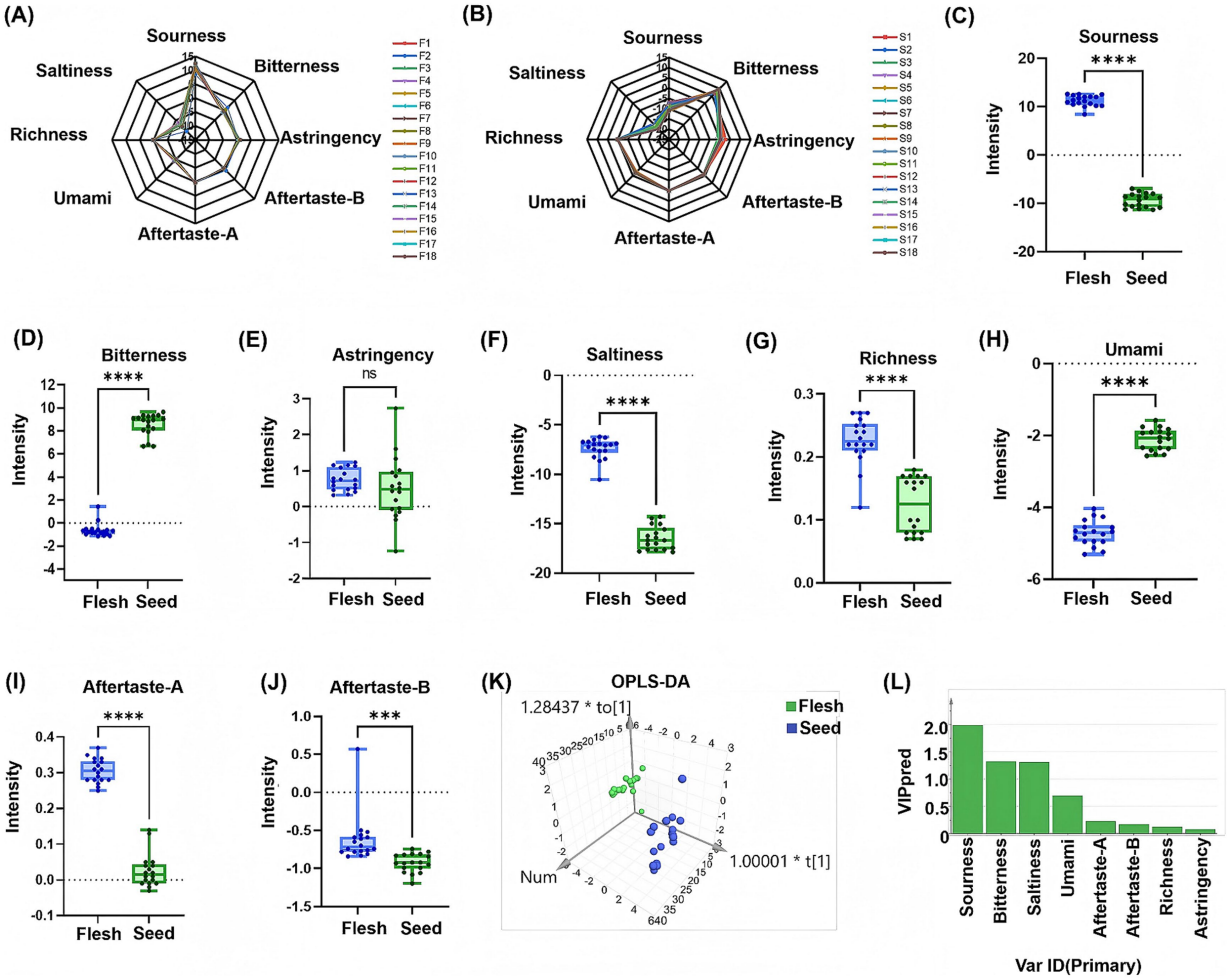
**(A)** Electronic tongue radar chart of flesh; **(B)** electronic tongue radar chart of seeds; **(C–J)** comparison of eight taste attributes between the flesh and seeds (ns indicates no significant difference; *indicates 0.01 < *p* ≤ 0.05; **indicates 0.001 < *p* ≤ 0.01; ***indicates 0.0001 < *p* ≤ 0.001; ****indicates *p* < 0.0001); **(K)** OPLS-DA score plot based on the electronic tongue; **(L)** VIP score plot of differential taste attributes between flesh and seeds.

To verify and supplement the flavor profile of the fruit of *Schisandra chinensis*, we employed an electronic tongue to analyze the taste characteristics of both the flesh and the seeds. Sensory evaluation results indicated that sourness and bitterness were the primary taste characteristics of the flesh and the seeds, respectively. The electronic tongue further confirmed the accuracy of these results. Typically, the sourness threshold for the electronic tongue was set at −13, and a detection value above this threshold was considered indicative of a sour taste. In the flesh (Figure 3A), sourness exhibited the highest detection value. The sourness values of the 18 flesh samples ranged from 8.44 to 12.64, with an average value of 11.26. This indicated that the flesh possessed notably strong sourness, which was consistent with the sensory evaluation results. The bitterness threshold of the electronic tongue was 0, and values above this threshold were considered to indicate bitterness. In the seeds (Figure 3B), the highest bitterness detection value was observed. The bitterness value of the 18 seed samples ranged from 6.66 to 9.64, with an average of 8.54. This indicated that the seeds of *Schisandra chinensis* possessed a strong bitterness. The above results indicated that the results of sensory evaluation and electronic tongue analysis were highly consistent in detecting sourness and bitterness of the fruit of *Schisandra chinensis*. Besides the tastes mentioned above, the electronic tongue could detect flavor profiles not captured by sensory evaluation. Based on the analysis of Figures 3A, B, combined with the comparison of different taste characteristics in the flesh and the seeds (Figures 3C–J), the following observations were made. The richness had an average value of 0.23 in the 18 flesh batches and 0.12 in the seed batches, indicating that the flesh had a higher richness. The astringent aftertaste (aftertaste-A) averaged 0.31 in the 18 flesh batches and 0.03 in the seed batches, demonstrating a more intense astringent aftertaste in the flesh. The detection values for bitter aftertaste (aftertaste-B) and umami in both the flesh and the seeds were below the threshold of 0, indicating that these taste attributes were not present in the flesh and the seeds. Overall, the results from the electronic tongue and sensory evaluation demonstrated both consistency and complementarity. It is noteworthy that the electronic tongue did not detect salty taste in either the flesh or seeds of *Schisandra chinensis*. This represented the primary difference between the electronic tongue and sensory evaluation. This is likely because the concentration of salty compounds in the fruit of *Schisandra chinensis* is relatively low, and the sensitivity of the electronic tongue is not as high as that of the human tongue.

Based on the aforementioned taste data, OPLS-DA was employed to further differentiate the flavor profiles of the flesh and seeds. To reveal the taste differences between the flesh and seeds, OPLS-DA (Figure 3K) was applied, which revealed complete separation between flesh and seed samples. In this model, the R^2^Y and Q^2^ values were 0.93 and 0.88, respectively, both approaching 1, which demonstrated the reliability of the model. Among the electronic taste values that exceeded the threshold (Figure 3L), sourness and bitterness were identified as key taste indicators for distinguishing flesh from seeds, with VIP values greater than 1.

Through comprehensive analysis of sensory evaluation and electronic tongue data, the distribution patterns of the prominent flavor characteristics of the fruit of *Schisandra chinensis* were established as follows: (1) strong sourness was predominantly distributed in the flesh, with only weak sourness detected in the seeds; (2) strong bitterness was mainly distributed in the seeds, with only weak bitterness detected in the flesh; (3) the lemon-like odor was more intense in the flesh than in the seeds; and (4) the intensities of pungency and Sichuan pepper odor were greater in the seeds than in the flesh.

### 3.4 LC-QTOF-MS/MS analysis

**Figure 4:**
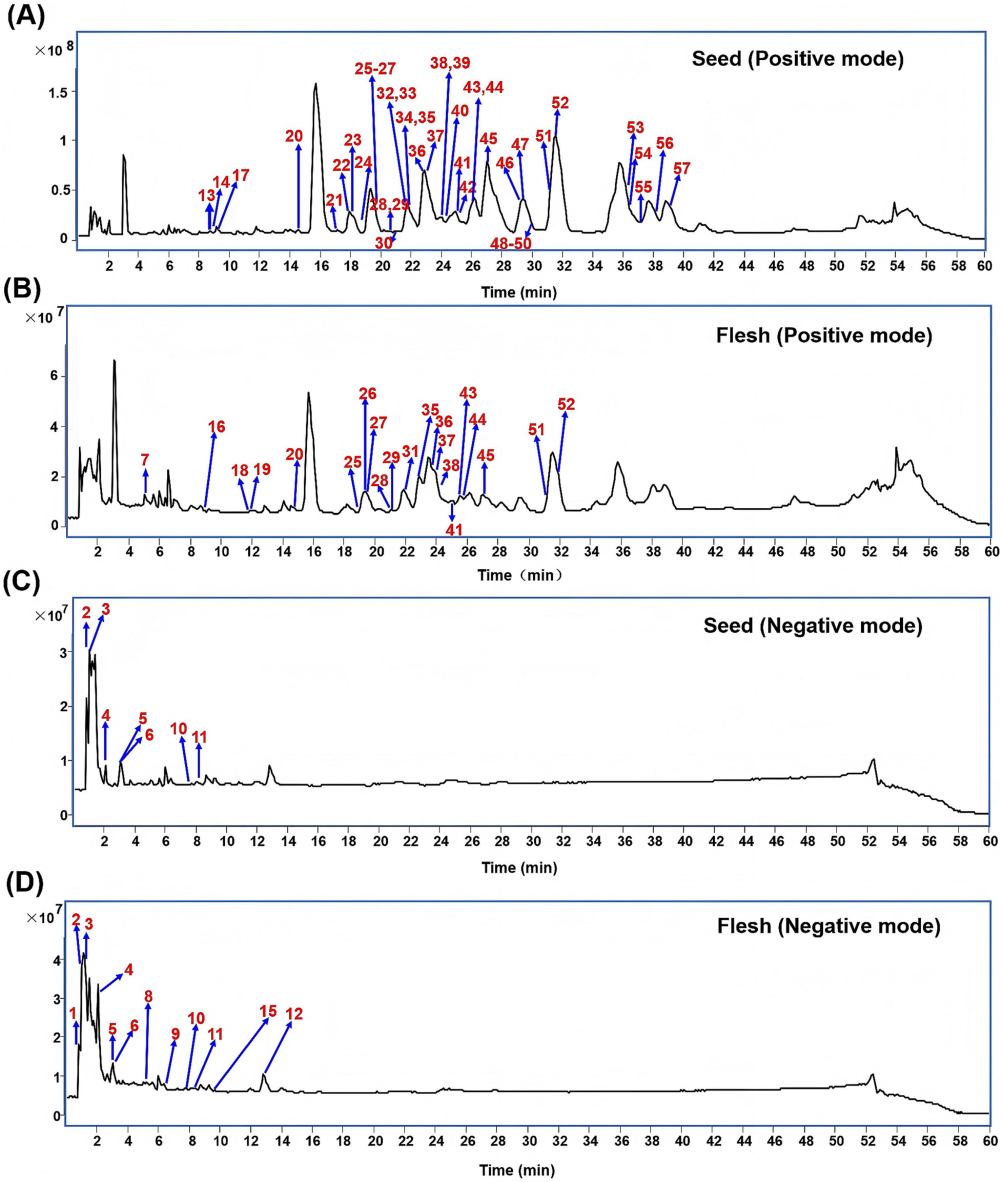
**(A)** Total ion chromatogram of seeds in positive ion mode; **(B)** Total ion chromatogram of flesh in positive ion mode; **(C)** Total ion chromatogram of seeds in negative ion mode; **(D)** Total ion chromatogram of flesh in negative ion mode.

To reveal the flavor components of *Schisandra chinensis* fruit, we used LC-QTOF-MS/MS to analyze the chemical constituents in its flesh and seeds. The total ion chromatograms of *Schisandra chinensis* flesh and seeds are presented in Figure 4. By evaluating retention time, mass spectrometric data, and tandem MS data, a total of 57 compounds were identified in both positive and negative ion modes, including 37 lignans, 12 terpenoids, 6 organic acids, 1 flavonoid, and 1 glycoside (Table 1). Specifically, the flesh contained 20 lignans, 8 terpenoids, 6 organic acids, 1 flavonoid, and 1 glycoside, while the seed contained 34 lignans, 5 terpenoids, and 5 organic acids. The mass deviations for all identified compounds were controlled within ±5 ppm, which ensured the accuracy of identification. The identification of ten compounds including schisandrin A, schisandrin B, schisandrol A, schisandrol B, schisantherin A, schisantherin B, schisanhenol, citric acid, malic acid, and protocatechuic acid was confirmed by comparison with chemical reference standards (Supplementary Figure S1). The fragmentation patterns of several representative compounds are as follows. Schisandrin A exhibited typical lignan fragmentation behavior. Its protonated molecular ion was detected at m/z 417 [M + H]^+^. Primary fragmentation arose from cleavage of partial bonds in the eight-membered ring, generating a neutral loss of C₅H₁₀ (70 Da) to form the fragment ion at m/z 347 [M + H − C₅H₁₀]^+^. Subsequent methoxy loss (–OCH₃, 31 Da) yielded the secondary fragment ion at m/z 316 [M + H − C₅H₁₀ − OCH₃]^+^ (32). Citric acid produced characteristic decarboxylation and dehydration fragments. Its deprotonated molecular ion was observed at m/z 191 [M − H]^−^. Dehydration first gave the ion at m/z 173 [M − H − H₂O]^−^, and subsequent CO₂ neutral loss from this ion generated the decarboxylated fragment at m/z 147 [M − H − H₂O − CO₂]^−^ (33). Lancifodilactone C generated diagnostic fragments via neutral loss of small molecules and lactone ring cleavage. Dehydration afforded the ion at m/z 525 [M − H − H₂O]^−^, while lactone ring opening triggered CO₂ loss to produce the fragment at m/z 499 [M − H − CO₂]^−^ (34). As a typical flavonoid detected in samples, rutin displayed a distinctive glycosidic cleavage pathway. Its protonated parent ion at m/z 611 [M + H]^+^ lost rhamnose (C₆H₁₀O₄, 146 Da) to form the ion at m/z 465. Further neutral loss of glucose (C₆H₁₀O₅, 162 Da) from this intermediate yielded the diagnostic fragment at m/z 303 (35). For the other 47 compounds, reasonable inferences were drawn by matching their information with a self-built *Schisandra chinensis* database (Supplementary Table S1) and literature data (34, 36–39).

**Table 1:** LC-QTOF-MS/MS characterization of non-volatile components in flesh and seeds.

No.	t_R_ /min	m/z (Error,ppm)	Formula	Ionization mode	Fragment ion (m/z)	Identification	Species	Distribution	bitter	sour
1	1.052	191.057 (−4.63)	C_7_H_12_O_6_	[M-H]^−^	191.0555	Quinic acid^b^	Organic acids	F	/	0.923
2	1.204	133.0137 (4.08)	C_4_H_6_O_5_	[M-H]^−^	115.0028	Malic acid^a^	Organic acids	S, F	/	0.999
3	1.339	191.0197 (0.14)	C_6_H_8_O_7_	[M-H]^−^	129.0180, 147.0269 173.0087	Citric acid^a^	Organic acids	S, F	/	0.999
4	2.156	205.0349 (2.31)	C_7_H_10_O_7_	[M-H]^−^	161.9819	6-methyl citrate^b^	Organic acids	S, F	0.566	0.621
5	3.005	153.0188 (3.46)	C_7_H_6_O_4_	[M-H]^−^	108.0211	Protocatechuic acid^a^	Organic acids	S, F	/	0.997
6	3.076	219.0506 (1.94)	C_8_H_12_O_7_	[M-H]^−^	175.0390	Dimethyl citrate^b^	Organic acids	S, F	0.730	0.570
7	5.288	611.1614 (0.31)	C_27_H_30_O_16_	[M + H]^+^	303.0506 465.1739	Rutin^b^	Flavonoids	F	/	/
8	5.644	463.0875 (1.51)	C_21_H_20_O_12_	[M-H]^−^	151.0033, 243.0279, 255.0299, 271.0237, 301.0332	Hyperoside^b^	Glycosides	F	/	/
9	6.316	611.2337 (1.47)	C_28_H_38_O_12_	[M + HCOO]^−^	373.1262	3,7-Dihydroxy-1,2,13,14-tetramethoxydibenzocyclooctadiene 12-O-*β*-D-glucopyranoside^b^	Lignans	F	/	/
10	7.787	559.2175 (1.76)	C_29_H_36_O_11_	[M-H]^−^	383.1487, 403.1519, 465.1514, 541.2069	Schindilactone C^b^	Terpenoids	S, F	/	/
11	8.011	575.2124 (1.74)	C_29_H_36_O_12_	[M-H]^−^	557.2008	Arisanlactone B^b^	Terpenoids	S, F	0.785	/
12	8.684	559.2169 (2.83)	C_29_H_36_O_11_	[M-H]^−^	347.1480, 413.1883	Schicagenin B^b^	Terpenoids	F	0.637	/
13	8.818	567.2202 (−0.24)	C_29_H_36_O_10_	[M + Na]^+^	508.2367	Pre-schisanartanin N^b^	Terpenoids	S	/	/
14	9.242	533.2383 (−0.33)	C_28_H_36_O_10_	[M + Na]^+^	341.1362, 401.1740	Wuweizidilactone H^b^	Terpenoids	S	0.881	/
15	9.243	543.2227 (1.6)	C_29_H_36_O_10_	[M-H]^−^	445.1906, 481.2207, 499.2284, 525.2082	Lancifodilactone C^b^	Terpenoids	F	0.771	/
16	9.414	571.2521 (2.94)	C_31_H_38_O_10_	[M + H]^+^	511.2324	Wuweizidilactone M^b^	Terpenoids	F	0.831	/
17	9.640	553.2044 (0.03)	C_28_H_34_O_10_	[M + Na]^+^	401.1611, 357.1348	Gomisin D^b^	Lignans	S	0.516	/
18	11.813	441.1888 (−1.02)	C_23_H_30_O_7_	[M + Na]^+^	204.8841	Gomisin S^b^	Lignans	F	/	/
19	12.053	441.1888 (−1.02)	C_23_H_30_O_7_	[M + Na]^+^	354.0756	Gomisin T^b^	Lignans	F	/	/
20	15.671	455.2038 (0.52)	C_24_H_32_O_7_	[M + Na]^+^	423.2249, 353.1754, 455.2052	Schisandrol A^a^	Lignans	S, F	/	/
21	17.303	549.2094 (0.2)	C_29_H_34_O_9_	[M + Na]^+^	509.2135	Lancifodilactone D^b^	Terpenoids	S	0.764	/
22	17.991	401.1605 (−2.55)	C_22_H_24_O_7_	[M + H]^+^	357.1341, 341.1017, 311.0905, 299.0903	Methyl 2,5-bis(3,4-dimethoxyphenyl)-4,5-dihydro-3-furancarboxylate^b^	Lignans	S	/	/
23	18.471	389.196 (−0.35)	C_22_H_28_O_6_	[M + H]^+^	279.0999, 317.1013, 327.1213, 342.1470	Gomisin J^b^	Lignans	S	/	/
24	18.559	561.2094 (0.19)	C_30_H_34_O_9_	[M + Na]^+^	417.1900	Schisantherin E^b^	Lignans	S	/	/
25	19.118	399.1802 (0.04)	C_23_H_26_O_6_	[M + H]^+^	301.1023	Allyl 2-O-benzoyl-3-O-benzyl-6-deoxy-*α*-L-mannopyranoside^b^	Lignans	S, F	/	/
26	19.335	455.147 (−0.81)	C_23_H_28_O_7_	[M + K]^+^	337.1422, 353.1355, 368.1608, 399.1790	Schisandrol B^a^	Lignans	S, F	/	/
27	19.526	455.1469 (−0.57)	C_23_H_28_O_7_	[M + K]^+^	315.0884, 330.1096, 353.1338	Epigomisin O^b^	Lignans	S, F	0.559	/
28	21.426	553.2409 (−3.01)	C_29_H_38_O_9_	[M + Na]^+^	372.1569, 387.1809	Angeloylgomisin Q^b^	Lignans	S, F	0.547	/
29	21.500	523.2303 (−0.12)	C_28_H_36_O_8_	[M + Na]^+^	353.136, 437.1538, 369.1679, 303.1176	Tigloylgomisin H^b^	Lignans	S, F	0.525	/
30	21.725	483.2378 (−0.15)	C_28_H_34_O_7_	[M + Na]^+^	247.0964, 349.1067, 379.1179, 367.1173, 437.1943, 395.1483	Gedunin^b^	Terpenoids	S	0.642	/
31	21.970	431.2079 (−3.42)	C_24_H_30_O_7_	[M + H]^+^	356.1625, 372.1577, 400.1880	Schinsanlignone A^b^	Lignans	F	/	/
32	22.068	523.2313 (−2.12)	C_28_H_36_O_8_	[M + Na]^+^	401.1589	Angeloylgomisin H^b^	Lignans	S	0.525	/
33	22.188	553.2411 (−0.56)	C_29_H_38_O_9_	[M + Na]^+^	399.1791	Pre-schisanartanin F^b^	Terpenoids	S	/	/
34	22.643	523.2323 (0.66)	C_30_H_34_O_8_	[M + H]^+^	315.1222, 386.1722, 395.1460, 409.1619, 441.1880	Benzoylgomisin H^b^	Lignans	S	/	/
35	22.789	401.1959 (−0.09)	C_23_H_28_O_6_	[M + H]^+^	300.0959, 386.1719, 370.1768, 316.093	*γ*-Schisandrol A^b^	Lignans	S, F	0.512	/
36	23.077	401.196 (−0.34)	C_23_H_28_O_6_	[M + H]^+^	295.1319, 323.1272, 355.1536, 370.1767	Rubschisandrin^b^	Lignans	S, F	0.512	/
37	24.297	537.21 (1.14)	C_30_H_32_O_9_	[M + H]^+^	310.1071, 325.1078, 340.1310, 356.1265, 371.1496	Gomisin G^b^	Lignans	S, F	0.509	/
38	24.300	537.2096 (−0.19)	C_28_H_34_O_9_	[M + Na]^+^	323.1291, 355.1512, 385.1604	Wuweizidilactone O^b^	Lignans	S	0.879	/
39	24.796	403.2117 (−0.46)	C_23_H_30_O_6_	[M + H]^+^	287.091, 301.1068, 341.1372, 371.1847	Gomisin K2^b^	Lignans	S	/	/
40	25.355	403.2115 (0.04)	C_23_H_30_O_6_	[M + H]^+^	317.1021, 331.117, 356.1610, 372.1921, 388.1879	Gomisin K1^b^	Lignans	S	/	/
41	25.835	415.1754 (−0.65)	C_23_H_26_O_7_	[M + H]^+^	315.0857, 355.1519	Neoisostegane^b^	Lignans	S, F	0.602	/
42	26.959	403.2117 (−0.46)	C_23_H_30_O_6_	[M + H]^+^	331.1181, 403.2116	Schisanhenol^a^	Lignans	S, F	/	/
43	27.395	553.1839 (−0.89)	C_28_H_34_O_9_	[M + K]^+^	385.1647, 415.1748	Schisantherin B^a^	Lignans	S, F	0.595	/
44	27.852	559.194 (−0.27)	C_30_H_32_O_9_	[M + Na]^+^	299.0907, 325.1072, 340.1300, 356.1251, 371.1485, 415.1748	Schisantherin A^a^	Lignans	S, F	0.509	/
45	29.459	515.2277 (−0.27)	C_28_H_34_O_9_	[M + H]^+^	355.1532, 316.0937, 312.0991	Schisantherin C^b^	Lignans	S	/	/
46	29.978	543.1629 (−0.67)	C_29_H_28_O_9_	[M + Na]^+^	399.1432, 368.1226, 341.1370	Schisantherin D^b^	Lignans	S	/	/
47	30.394	219.1742 (0.65)	C_15_H_22_O	[M + H]^+^	175.1469	Chamigrenal^b^	Terpenoids	S	0.644	/
48	30.729	409.1622 (−0.1)	C_22_H_26_O_6_	[M + Na]^+^	368.3921	Gomisin M2^b^	Lignans	S	0.549	/
49	30.969	409.1623 (−0.36)	C_22_H_26_O_6_	[M + Na]^+^	311.1418	Gomisin L1^b^	Lignans	S	0.549	/
50	31.122	417.2271 (0.16)	C_24_H_32_O_6_	[M + H]^+^	316.1310, 347.1495, 370.1771, 386.2084, 402.2035	Schisandrin A^a^	Lignans	S, F	0.603	/
51	31.969	417.2283 (−2.73)	C_24_H_32_O_6_	[M + H]^+^	301.1073, 316.1303, 347.1484, 402.2035	Deoxyschisandrin^b^	Lignans	S, F	0.603	/
52	35.846	401.196 (−0.34)	C_23_H_28_O_6_	[M + H]^+^	316.0951, 354.1458, 370.1768, 386.1726	Schisandrin B^a^	Lignans	S, F	0.512	/
53	36.775	521.215 (3.83)	C_30_H_32_O_8_	[M + H]^+^	399.1795, 368.1612, 330.1092, 315.0864, 299.0907, 285.0764	Benzoylisogomisin O^b^	Lignans	S	0.567	/
54	36.888	519.1999 (2.79)	C_30_H_30_O_8_	[M + H]^+^	181.0854, 343.1168	Schisanchinin A^b^	Lignans	S	/	/
55	37.063	521.2149 (4.03)	C_30_H_32_O_8_	[M + H]^+^	399.1793, 384.1562, 368.1611, 341.1740	6-O-benzoylgomisin O^b^	Lignans	S	0.567	/
56	38.511	543.1989 (0.08)	C_30_H_32_O_8_	[M + Na]^+^	384.1562, 369.1681, 399.1798,	Benzoylgomisin O^b^	Lignans	S	0.567	/
57	39.487	537.1894 (−1.75)	C_28_H_34_O_8_	[M + K]^+^	239.0709, 357.1324	Angeloylgomisin O^b^	Lignans	S	0.612	/

a The compounds were identified by comparing with reference substances.

b The compounds were tentatively identified by matching data from the self-established database and published literature.

F, flesh; S, seed. Bitter and sour values were predicted by VirtualTaste.

**Figure 5:**
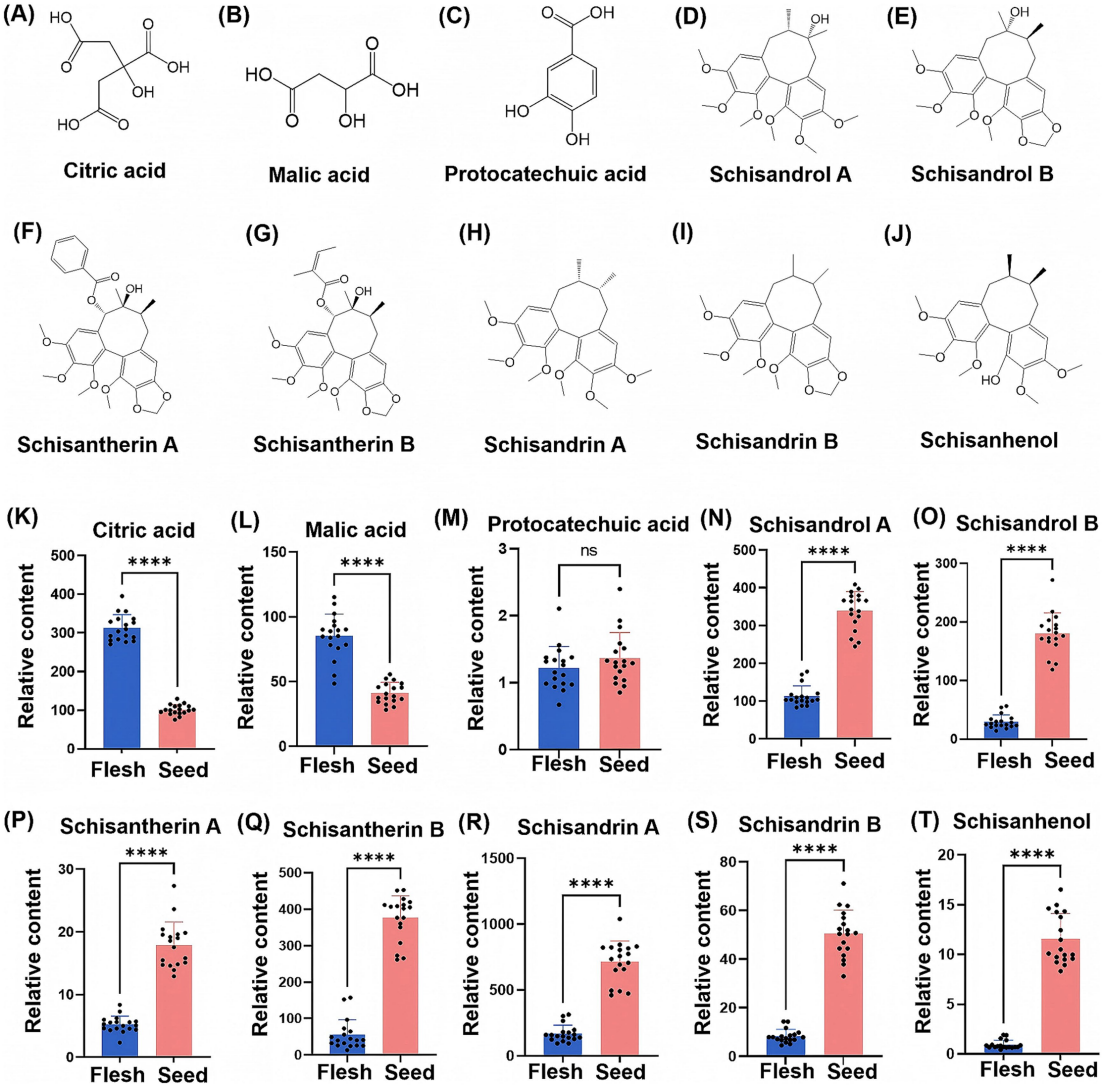
Chemical structures of potential taste components including **(A)** citric acid; **(B)** malic acid; **(C)** protocatechuic acid; **(D)** schisandrol A; **(E)** schisandrol B; **(F)** schisantherin A; **(G)** schisantherin B; **(H)** schisandrin A; **(I)** schisandrin B; and **(J)** schisanhenol. **(K–T)** *Comp*arison of relative contents of these ten compounds between the flesh and seeds (ns indicates no significant difference; *indicates 0.01 < *p* ≤ 0.05; **indicates 0.001 < *p* ≤ 0.01; ***indicates 0.0001 < *p* ≤ 0.001; ****indicates *p* < 0.0001).

To compare flavor components between the flesh and seeds, an analysis of the relative contents of 10 compounds (Figures 5A–J) was conducted with chemical reference standards. The relative contents of citric acid (Figure 5K) and malic acid (Figure 5L) showed differences between the flesh and seeds, with markedly higher levels in the flesh, whereas protocatechuic acid (Figure 5M) demonstrated no significant difference in relative content between the two parts. Although these three acidic compounds were present in both the flesh and seeds, their differences in relative contents suggested that sourness was more prominent in the flesh. This finding was consistent with electronic tongue data, which identified sourness as the predominant taste in the flesh. Furthermore, seven lignans including schisandrol A (Figure 5N), schisandrol B (Figure 5O), schisantherin A (Figure 5P), schisantherin B (Figure 5Q), schisandrin A (Figure 5R), schisandrin B (Figure 5S), and schisanhenol (Figure 5T) showed significant differences (*p* < 0.0001) in relative content between the flesh and seeds, with markedly higher levels in the seeds. Notably, these lignans are widely considered to be the functional constituents of *Schisandra chinensis* (40).

### 3.5 Prediction of taste by VirtualTaste

To elucidate the flavor differences between the flesh and seeds, the VirtualTaste platform built on a random forest algorithm was adopted to predict the taste-type classification probabilities of compounds identified via LC-QTOF-MS/MS (Table 1). The results demonstrated that a total of 31 compounds were predicted to possess bitter taste attributes. Among these, wuweizidilactone M, lancifodilactone C, and schicagenin B were exclusively present in the flesh, whereas benzoylgomisin O, schisantherin C, 6-O-benzoylgomisin O, angeloylgomisin H, gedunin, gomisin D, benzoylisogomisin O, angeloylgomisin O, gomisin L1, lancifodilactone D, wuweizidilactone H, wuweizidilactone O, and chamigrenal were found only in the seeds. An additional 15 compounds with bitter taste attribution were simultaneously present in both the flesh and seeds, including schisantherin A, schisantherin B, tigloylgomisin H, angeloylgomisin Q, schisandrin A, schisandrin B, gomisin G, *γ*-schisandrol A, epigomisin O, rubschisandrin, neoisostegane, deoxyschisandrin, 6-methyl citrate, dimethyl citrate, and arisanlactone B. The seeds contained a total of 28 compounds with bitter taste attribution, which were more numerous than the 20 detected in the flesh, a finding that aligned with electronic tongue results identifying bitterness as the most prominent taste in the seeds. The prediction for schisandrol A (Figure 5D), schisandrol B (Figure 5E), and schisanhenol (Figure 5J) indicated an absence of bitterness. We further predicted the sour taste attribution probabilities of six acidic components, including citric acid, malic acid, protocatechuic acid, quinic acid, 6-methyl citrate, and dimethyl citrate. Five of these organic acids were present in both the flesh and the seeds. The sour taste classification probabilities of citric acid and malic acid both reached 0.999, indicating an extremely high likelihood that both compounds belonged to the sour taste category. The contents of these two compounds were higher in the flesh compared to the seeds, which were consistent with the detection results from the electronic tongue. The prediction for protocatechuic acid was 0.997, which was lower than that of both citric acid and malic acid. Moreover, its content showed no significant difference between the flesh and seeds (Figure 5M), indicating that protocatechuic acid was not the main contributor to the difference in sourness between the flesh and seeds of *Schisandra chinensis*. According to existing literature, the three dissociation constants of citric acid were 3.13, 4.76, and 6.40, the two dissociation constants of malic acid were 3.51 and 5.03, and the dissociation constant of protocatechuic acid was 4.48 (41–43). These data supported the high reliability of the VirtualTaste model in predicting sourness. The remaining three acids displayed gradually declining sour classification probabilities: quinic acid (0.923), 6-methyl citrate (0.621), and dimethyl citrate (0.570). It was worth noting that the VirtualTaste platform was only capable of predicting bitter and sour components in *Schisandra chinensis*. Other flavor substances, such as pungency and Sichuan pepper odor, were identified via HS-GC-IMS combined with aroma database matching.

In summary, the flesh exhibited a more sour taste due to its higher content of citric acid and malic acid, while the seeds contained both a greater diversity of bitter-tasting lignans and higher concentrations of these compounds. This finding provided a clear explanation for the differential distribution of sourness and bitterness observed in both sensory evaluation and electronic tongue analysis. It was noteworthy that the majority of bitter components and functional constituents in the seeds were lignans. Such results indicated that some bitter lignans with biological activities should not be readily removed during the processing of *Schisandra chinensis* to avoid the loss of functional constituents.

### 3.6 Quantitative analysis of bitter and functional components

**Figure 6:**
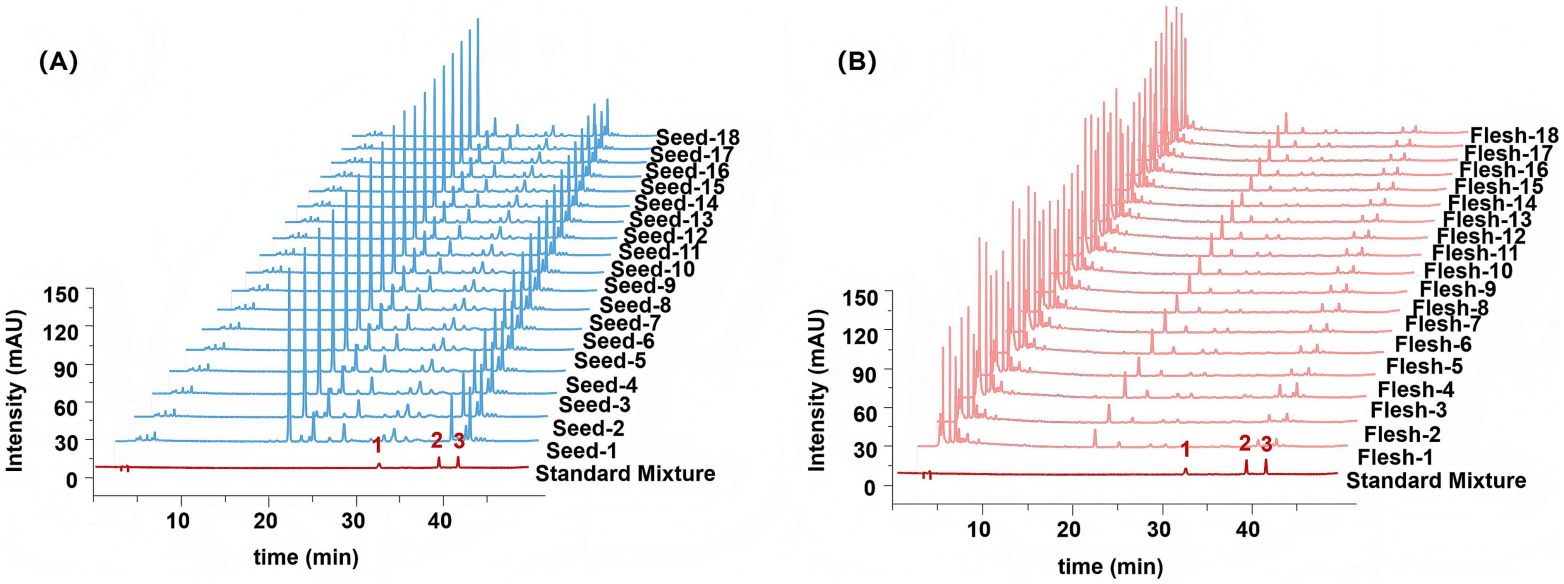
**(A)** Chromatographic fingerprints of 18 batches of *Schisandra chinensis* seeds. **(B)** Chromatographic fingerprints of 18 batches of *Schisandra chinensis* flesh. Peak 1: Schisantherin A; Peak 2: Schisandrin A; Peak 3: Schisandrin B.

Schisantherin A, schisandrin A and schisandrin B were predicted to be potential bitter constituents and further confirmed as functional components of *Schisandra chinensis*. Therefore, HPLC was employed to quantify their contents in the seeds and flesh. The HPLC chromatograms of the 18 batches of samples (Figure 6) showed satisfactory separation of the characteristic peaks, thus satisfying the criteria for quantitative analysis. All reference standards exhibited good linearity within the established concentration ranges (Supplementary Figure S2), and sufficiently low limits of detection and quantification were achieved (Table 2). Methodological validation demonstrated that the spiked recoveries ranged from 104.07 to 106.56%, with RSD values below 1.23% (Table 3). No interference from the blank matrix was observed (Supplementary Figure S3). The inter-day and intra-day precisions were also evaluated, yielding RSD values of less than 1.8% (Supplementary Tables S2 and S3). Under the experimental conditions, the matrix effects in the flesh and seeds were less than 5% (Supplementary Tables S4 and S5). These results collectively confirmed the accuracy and reliability of the quantitative method. The quantitative results were summarized in Table 4. In the 18 batches of seed samples, the contents of schisantherin A, schisandrin A, and schisandrin B were in the ranges of 2.24–3.61, 2.91–4.06, and 3.23–4.33 mg/g, respectively. Schisantherin A was present at extremely low concentrations in flesh, and its chromatographic signal failed to meet the detection limit of the instrument. The contents of schisandrin A and schisandrin B in flesh across the 18 batches ranged from 0.23 to 0.37 mg/g and from 0.34 to 0.47 mg/g, respectively. Overall, the levels of the three lignans in flesh were lower than those in seeds. These three lignans were speculated to be key bitter-active constituents. Their higher concentrations in seeds relative to flesh might partly explain the stronger bitter taste of seeds compared with flesh.

**Table 2:** LOD and LOQ for Schisantherin A, Schisandrin A, and Schisandrin B.

Analytes	LOD (μg/mL)	LOQ (μg/mL)
Schisantherin A	0.905	3.018
Schisandrin A	0.651	2.169
Schisandrin B	0.621	2.071

**Table 3:** Spiked recoveries of schisantherin A, schisandrin A, and schisandrin B.

Sample	Native content (mg)	Spiked standard (mg)	Detected total (mg)	Recovery rate (%)	Average recovery rate (%)	RSD (%)
Schisantherin A	0.1149	0.2913	0.4245	106.28	106.56	0.41
	0.1144	0.2913	0.4231	105.97		
	0.1147	0.2913	0.4244	106.32		
	0.1146	0.2913	0.4268	107.17		
	0.1143	0.2913	0.4254	106.80		
	0.1148	0.2913	0.4259	106.80		
Schisandrin A	0.2402	0.2322	0.4871	106.33	104.07	1.23
	0.2458	0.2322	0.4865	103.66		
	0.2425	0.2322	0.4853	104.57		
	0.2437	0.2322	0.4829	103.01		
	0.2422	0.2322	0.4838	104.05		
	0.2495	0.2322	0.4882	102.80		
Schisandrin B	0.3585	0.2754	0.6405	102.40	104.18	1.02
	0.3542	0.2754	0.6409	104.10		
	0.3527	0.2754	0.6406	104.54		
	0.3569	0.2754	0.6435	104.07		
	0.3517	0.2754	0.6428	105.70		
	0.3551	0.2754	0.6422	104.25		

**Table 4:** Contents of schisantherin A, schisandrin A, and schisandrin B in flesh and seeds.

No.	Schisantherin A (mg/g)		Schisandrin A (mg/g)		Schisandrin B (mg/g)	
	Flesh	Seed	Flesh	Seed	Flesh	Seed
1	ND	2.96	0.32	3.09	0.44	4.33
2	ND	2.98	0.31	3.08	0.47	4.30
3	ND	2.98	0.30	3.07	0.43	4.27
4	ND	2.63	0.38	3.38	0.39	3.87
5	ND	2.58	0.29	3.26	0.41	3.74
6	ND	2.41	0.33	3.10	0.45	3.55
7	ND	2.33	0.28	2.99	0.38	3.42
8	ND	2.27	0.30	2.91	0.34	3.34
9	ND	2.67	0.29	3.37	0.45	3.77
10	ND	2.58	0.30	3.24	0.45	3.60
11	ND	2.42	0.23	3.04	0.38	3.38
12	ND	2.46	0.31	3.08	0.44	3.41
13	ND	2.38	0.32	2.93	0.46	3.23
14	ND	2.24	0.36	3.42	0.42	3.51
15	ND	3.36	0.31	3.84	0.43	3.93
16	ND	3.61	0.30	4.06	0.44	4.17
17	ND	3.27	0.29	3.70	0.41	3.79
18	ND	3.26	0.27	3.71	0.43	3.80

ND, not detected (below LOD).

### 3.7 HS-GC-IMS analysis

**Figure 7:**
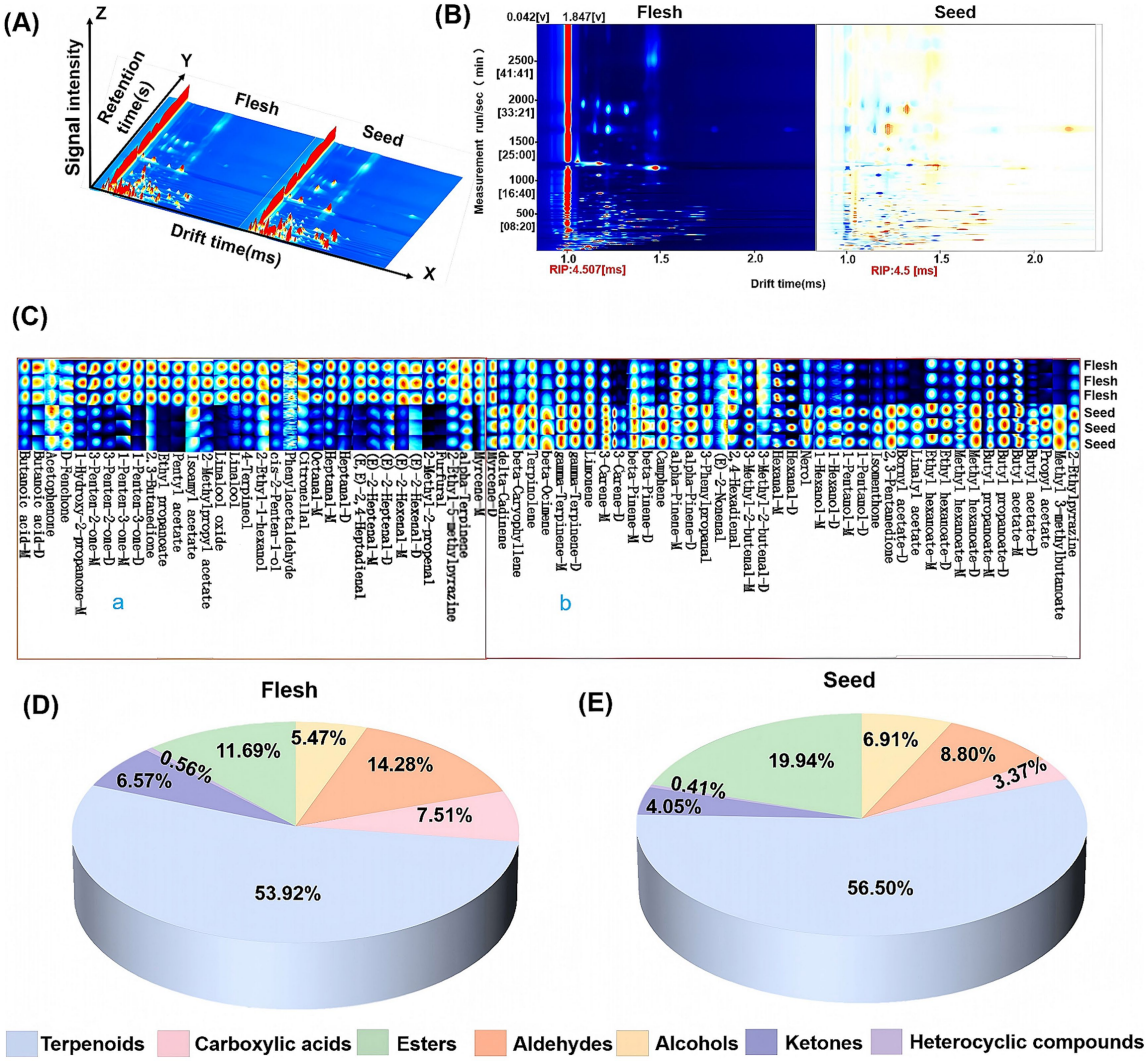
Comparative analysis of volatile components in the flesh and seeds based on HS-GC-IMS. **(A)** 3D topographic plots of flesh and seeds. **(B)** 2D difference plots of flesh and seeds. Red dots represent compounds with higher relative abundance in seeds, and blue dots represent those with higher relative abundance in flesh; White areas indicate no significant difference between the two groups. **(C)** Comparison of relative contents of volatile components in flesh and seeds. Region a represents components enriched in flesh, while region b represents those enriched in seeds. **(D)** Proportional distribution of volatile compound classes in flesh. **(E)** Proportional distribution of volatile compound classes in seeds.

HS-GC-IMS was employed to investigate the differences in pungent components between the flesh and seeds of *Schisandra chinensis*. The results revealed that a total of 76 volatile compounds were identified in the flesh and seeds of *Schisandra chinensis*, encompassing 20 terpenes, 18 aldehydes, 16 esters, 10 ketones, 8 alcohols, 2 carboxylic acids, and 2 heterocyclic compounds (Table 5). The three-dimensional HS-GC-IMS spectra of the flesh and seeds are presented in Figure 7A, with the X-axis, Y-axis, and Z-axis representing drift time, retention time, and signal peak intensity, respectively. To further reveal the differences in volatile compounds between the flesh and seeds, the flesh spectrum was selected as the reference. A differential comparison plot was generated by subtracting the reference standard from the seed spectrum (Figure 7B). In this plot, the background appeared white after subtraction when the relative content of a particular volatile compound was comparable between the flesh and seeds. Red regions indicated that the concentration of a compound was higher in the seeds than in the flesh, while blue regions signified that the concentration was lower in the seeds than in the flesh. The differential composition between the flesh and seeds is displayed in Figure 7C. In this spectrum, brighter spots represented higher concentrations. In region a, 34 volatile compounds showed higher concentrations in the flesh than in the seeds, while in region b, 42 volatile compounds exhibited higher concentrations in the seeds than in the flesh. Among the 76 identified volatile components, nerol, limonene and linalool exhibited the characteristic lemon-like odor (44, 45). Ten pungent components were identified, including *γ*-terpinene-M, *γ*-terpinene-D, 3-carene-M, 3-carene-D, *β*-pinene-M, *β*-pinene-D, camphene, *α*-pinene-M, *α*-pinene-D, and *β*-caryophyllene (46–49). All ten of these pungent components showed higher concentrations in the seeds. Myrcene-D and linalyl acetate contributed to the characteristic odor of Sichuan pepper (50). Similarly, these two compounds were present at higher levels in the seeds. This finding was consistent with the results of sensory evaluation, providing a robust explanation for the stronger pungency in the seeds compared to the flesh. In short, the material basis of the lemon-like odor, Sichuan pepper odor, and pungent taste of the fruit of *Schisandra chinensis* was uncovered.

**Table 5:** HS-GC-IMS analysis of volatile compounds in flesh and seeds.

No.	Compound	Formula	MW	RI	Rt (s)	Dt (RIPrel)
1	2-Methyl-2-propenal	C_4_H_6_O	70.1	892.1	286.08	1.21668
2	Ethyl propanoate	C_5_H_10_O_2_	102.1	964.3	336.711	1.46721
3	Propyl acetate	C_5_H_10_O_2_	102.1	989	355.978	1.47646
4	2,3-Butanedione	C_4_H_6_O_2_	86.1	991	357.624	1.17754
5	Methyl 3-methylbutanoate	C_6_H_12_O_2_	116.2	1018.7	386.181	1.2141
6	2-Methylpropyl acetate	C_6_H_12_O_2_	116.2	1025.2	393.51	1.23481
7	alpha-Pinene-M	C_10_H_16_	136.2	1032.6	401.925	1.21556
8	alpha-Pinene-D	C_10_H_16_	136.2	1032.8	402.201	1.66406
9	1-Penten-3-one-M	C_5_H_8_O	84.1	1037.8	407.979	1.08023
10	1-Penten-3-one-D	C_5_H_8_O	84.1	1,038	408.254	1.31063
11	Camphene	C_10_H_16_	136.2	1060.6	435.771	1.20102
12	2,3-Pentanedione	C_5_H_8_O_2_	100.1	1071.5	449.709	1.2131
13	Butyl acetate-M	C_6_H_12_O_2_	116.2	1083.9	465.997	1.25268
14	Butyl acetate-D	C_6_H_12_O_2_	116.2	1084.6	466.955	1.61825
15	Hexanal-D	C_6_H_12_O	100.2	1097.4	484.68	1.56353
16	Hexanal-M	C_6_H_12_O	100.2	1097.7	485.159	1.27829
17	beta-Pinene-M	C_10_H_16_	136.2	1114.3	512.465	1.21193
18	beta-Pinene-D	C_10_H_16_	136.2	1114.3	512.465	1.21193
19	3-Carene-M	C_10_H_16_	136.2	1,130	539.77	1.21426
20	3-Carene-D	C_10_H_16_	136.2	1130.1	539.835	1.72557
21	Isoamyl acetate	C_7_H_14_O_2_	130.2	1134.9	548.52	1.30243
22	3-Penten-2-one-M	C_5_H_8_O	84.1	1143.9	565.16	1.07688
23	3-Penten-2-one-D	C_5_H_8_O	84.1	1144.2	565.639	1.34233
24	Butyl propanoate-M	C_7_H_14_O_2_	130.2	1152.8	581.927	1.28644
25	Butyl propanoate-D	C_7_H_14_O_2_	130.2	1153.5	583.364	1.72186
26	Myrcene-M	C_10_H_16_	136.2	1174.7	625.52	1.21426
27	Myrcene-D	C_10_H_16_	136.2	1174.8	625.766	1.71395
28	Pentyl acetate	C_7_H_14_O_2_	130.2	1182.8	642.626	1.31824
29	alpha-Terpinene	C_10_H_16_	136.2	1184.9	647.078	1.21426
30	Methyl hexanoate-D	C_7_H_14_O_2_	130.2	1186.5	650.431	1.68927
31	Methyl hexanoate-M	C_7_H_14_O_2_	130.2	1186.9	651.389	1.26083
32	Heptanal-M	C_7_H_14_O	114.2	1193.5	664.323	1.35164
33	Heptanal-D	C_7_H_14_O	114.2	1194.2	665.282	1.69392
34	Limonene	C_10_H_16_	136.2	1202.8	677.021	1.21762
35	3-Methyl-2-butenal-M	C_5_H_8_O	84.1	1212.1	689.713	1.09085
36	3-Methyl-1-butanol-D	C_5_H_12_O	88.1	1218.6	698.834	1.49338
37	3-Methyl-1-butanol-M	C_5_H_12_O	88.1	1,219	699.461	1.24598
38	Ethyl hexanoate-M	C_8_H_16_O_2_	144.2	1225.6	708.863	1.34417
39	Ethyl hexanoate-D	C_8_H_16_O_2_	144.2	1226.3	709.765	1.82233
40	(E)-2-Hexenal-M	C_6_H_10_O	98.1	1229.6	714.505	1.18221
41	(E)-2-Hexenal-D	C_6_H_10_O	98.1	1230.4	715.758	1.52144
42	gamma-Terpinene-M	C_10_H_16_	136.2	1250.9	745.846	1.21665
43	gamma-Terpinene-D	C_10_H_16_	136.2	1250.9	745.846	1.70253
44	beta-Ocimene	C_10_H_16_	136.2	1261.8	762.418	1.2147
45	1-Pentanol-M	C_5_H_12_O	88.1	1262.4	763.396	1.25746
46	1-Pentanol-D	C_5_H_12_O	88.1	1262.4	763.396	1.51124
47	Terpinolene	C_10_H_16_	136.2	1,287	802.259	1.22047
48	Octanal-M	C_8_H_16_O	128.2	1,299	822.317	1.42069
49	1-Hydroxy-2-propanone-M	C_3_H_6_O_2_	74.1	1312.5	846.623	1.08016
50	(E)-2-Heptenal-M	C_7_H_12_O	112.2	1330.3	879.688	1.2597
51	(E)-2-Heptenal-D	C_7_H_12_O	112.2	1330.3	879.688	1.67069
52	cis-2-Penten-1-ol	C_5_H_10_O	86.1	1335.9	890.314	0.9484
53	2-Ethylpyrazine	C_6_H_8_N_2_	108.1	1358.9	935.556	1.14785
54	1-Hexanol-D	C_6_H_14_O	102.2	1368.4	955.045	1.64263
55	1-Hexanol-M	C_6_H_14_O	102.2	1368.8	955.814	1.33685
56	2,4-Hexadienal	C_6_H_8_O	96.1	1384.2	988.1	1.11242
57	D-Fenchone	C_10_H_16_O	152.2	1417.4	1061.278	1.29963
58	2-Ethyl-5-methylpyrazine	C_7_H_10_N_2_	122.2	1432.1	1095.464	1.20881
59	Linalool oxide	C_10_H_18_O_2_	170.3	1450.5	1139.757	1.25551
60	Furfural	C_5_H_4_O_2_	96.1	1,495	1254.381	1.33738
61	Citronellal	C_10_H_18_O	154.3	1497.4	1260.86	1.22688
62	Isomenthone	C_10_H_18_O	154.3	1516.3	1313.366	1.35225
63	Linalyl acetate	C_12_H_20_O_2_	196.3	1560.8	1445.447	1.22417
64	(E)-2-Nonenal	C_9_H_16_O	140.2	1576.1	1494.111	1.41275
65	(E, E)-2,4-Heptadienal	C_7_H_10_O	110.2	1576.7	1495.913	1.18326
66	Linalool	C_10_H_18_O	154.3	1632.3	1686.399	1.78317
67	Bornyl acetate-D	C_12_H_20_O_2_	196.3	1632.6	1687.439	2.1819
68	4-Terpineol	C_10_H_18_O	154.3	1695.3	1931.349	1.22154
69	Butanoic acid-M	C_4_H_8_O_2_	88.1	1710.1	1993.889	1.1651
70	Butanoic acid-D	C_4_H_8_O_2_	88.1	1710.6	1996.269	1.37037
71	beta-Caryophyllene	C_15_H_24_	204.4	1754.8	2195.912	1.42236
72	Phenylacetaldehyde	C_8_H_8_O	120.2	1766.4	2251.135	1.26502
73	Acetophenone	C_8_H_8_O	120.2	1813	2489.001	1.17749
74	delta-Cadinene	C_15_H_24_	204.4	1822	2537.643	1.44041
75	Nerol	C_10_H_18_O	154.3	1870.3	2815.964	1.2221
76	3-Phenylpropanal	C_9_H_10_O	134.2	1881.8	2886.554	1.10428

M and D represent monomer and dimer of the compound, respectively. MW, molecular weight; RI, retention index; Rt, retention time; Dt, drift time; RIPrel, drift time normalized relative to the reaction ion peak.

The proportional distributions of volatile components in the flesh and seeds are presented in Figures 7D,E, respectively. Terpenoids accounted for 53.92 and 56.50% of the total volatiles in the flesh and seeds, respectively, revealing that they were the dominant volatile constituents of *Schisandra chinensis*. Terpenoids were highly volatile and generally delivered characteristic aromas including citrus freshness, floral notes and woody odors. Esters and aldehydes also occupied relatively high proportions of the total volatile compounds. In particular, esters made up 19.94% of the volatiles in the seeds, a value higher than the 11.69% in the flesh. Esters mainly produced fruity or distinctive aromatic notes. Aldehydes, by contrast, constituted 14.28% of the volatiles in the flesh versus 8.80% in the seeds, and they endowed *Schisandra chinensis* with its unique fresh aroma. As the three most abundant classes of volatile compounds in the fruit of *Schisandra chinensis*, terpenoids, esters and aldehydes jointly constructed the complex flavor profile of the fruit via their individual characteristic odors.

## 4 Discussion

The flavor profiles and tissue-specific distributions of compounds in the flesh and seeds of *Schisandra chinensis* were analyzed in this study using a combination of taste and odor metabolomics. A variety of detection technologies were integrated in the research, including sensory evaluation, electronic tongue, LC-QTOF-MS/MS, VirtualTaste, and HS-GC-IMS. The respective advantages of different technologies were combined to effectively address the limitations of single detection methods, and a comprehensive analytical scheme was provided for the characterization of flavor substances in fruits with both medicinal and edible values. Although research on the flavor of *Schisandra chinensis* has gradually expanded, most existing studies have taken intact fruits as the research subject and overlooked the flavor differences between different tissues. Wang et al. (3) distinguished the gustatory characteristics of raw *Schisandra chinensis* and vinegar-processed *Schisandra chinensis* by electronic tongue technology. Sun et al. (51) adopted headspace-gas chromatography-ion mobility spectrometry to explore the influences of harvest time on volatile flavor components of *Schisandra chinensis*. Yan et al. (52) analyzed the correlation between fruit color differences and aroma components of *Schisandra chinensis* based on gas chromatography-ion mobility spectrometry. All the above studies conducted analyses solely on whole fruits and failed to characterize the differential accumulation patterns of flavor compounds between flesh and seed tissues. The present work characterizes the tissue-specific flavor traits of *Schisandra chinensis* flesh and seeds, filling the research gap left by prior whole-fruit-focused investigations.

Comprehensive analysis showed that the flavor characteristics of *Schisandra chinensis* flesh and seeds differed, and this difference probably arose from distinct biosynthetic pathways. Analysis of organic acid biosynthetic pathways demonstrates that citric acid is generated through the mitochondrial tricarboxylic acid (TCA) cycle. Malic acid, by contrast, is synthesized via two independent routes: the TCA cycle and a cytoplasmic parallel reaction catalyzed by malate dehydrogenase (MDH) (53). Based on these findings, we inferred that the biosynthetic pathways of citric acid and malic acid were more active in the flesh of *Schisandra chinensis*, leading to substantial accumulation of organic acids. Combined with the flavor prediction results from VirtualTaste, both citric acid and malic acid were identified as potential sour-tasting compounds. This likely served as the main reason for the strong sour taste in *Schisandra chinensis* flesh. Lignan biosynthesis takes phenylalanine derived from the shikimate pathway as the initial substrate. After coniferyl alcohol was synthesized through the phenylpropane pathway, various specific modification reactions including oxidative coupling, reduction, methylation, and glycosylation were carried out to form different lignan monomers and their derivatives (54). Lignan biosynthetic activity was higher in seeds than in flesh. According to the prediction results of VirtualTaste, multiple lignans were identified as potential bitter compounds, which likely accounted for the bitter flavor of *Schisandra chinensis* seeds.

This study clarified the distribution of flavor compounds and bioactive ingredients across different tissues of *Schisandra chinensis* fruits, providing a basis for improved processing and product development. At present, most manufacturers processing *Schisandra chinensis* adopt whole fruits as raw materials, overlooking the differences in chemical composition and sensory flavor between the flesh and seeds. Judging from existing research, Zhao et al. (55) explored the application of intact *Schisandra chinensis* fruits in anti-photoaging products, while Sergienko et al. (56) also carried out research on concentrated candied snacks of *Schisandra chinensis* with whole fruits as raw materials. None of the above studies distinguished the properties of different fruit tissues, resulting in a single processing strategy. In addition, we observed partial overlap between bitter compounds and core bioactive components of *Schisandra chinensis*, indicating that bitter taste cannot be completely removed via conventional treatments including physical tissue separation, simple decolorization, and conventional debittering processes. In response to this characteristic, a differentiated tissue-specific development strategy was adopted: the flesh accumulates abundant organic acids yet exhibits low sweetness, with excessive sourness as its sensory defect. Relying on existing fruit and vegetable processing technologies, its flavor was optimized through biological deacidification and moderate sweetener compounding to suit the development of common foods such as fruit juices, jams and fruit wines. Seeds are abundant in bioactive lignans and possess promising application prospects in the field of functional foods, with liver-protective, antioxidant and other health-care effects. On the premise of retaining active components, flavor masking technologies were applied to attenuate bitterness and address undesirable sensory drawbacks during consumption. This tissue-separated development mode enabled more rational utilization of the resource value of different tissues of *Schisandra chinensis*.

Although this study illustrated the tissue distribution characteristics of flavor compounds and functional components in the fruits of *Schisandra chinensis*, this work still contained several limitations. First, we did not investigate the changes in the composition and content of flavor substances or the sensory characteristics of the flesh and seeds under different drying and storage conditions. Second, this study only revealed the chemical basis for tissue-specific flavor divergence, without identifying the key structural genes and core metabolic pathways that regulate differential flavor formation between the flesh and seeds. Third, we did not perform *in vitro* cell assays or *in vivo* animal trials, and the dose-effect correlations of flavor-active compounds remain uncharacterized. Subsequent work will address the three limitations by exploring drying/storage-induced flavor variations, deploying multi-omics to dissect key metabolic pathways, and implementing *in vitro* and *in vivo* assays to clarify dose-effect relationships of flavor-active compounds.

## 5 Conclusion

In summary, this study developed an integrated approach combining sensory evaluation, electronic tongue, LC-QTOF-MS/MS, VirtualTaste, and HS-GC-IMS. By integrating taste and odor metabolomics, the work revealed the differential distribution of flavor compounds between *Schisandra chinensis* flesh and seeds. A total of 57 non-volatile taste-related compounds and 76 volatile aroma compounds were identified. The results demonstrated that sourness was predominantly distributed in the flesh, resulting from the high levels of citric acid and malic acid. In contrast, bitterness, pungency, and Sichuan pepper odor were mainly concentrated in the seeds. This distribution was ascribed to the higher abundance and diversity of lignans and terpenes in the seeds. Notably, the potentially bitter lignans in the seeds were also the main functional components of *Schisandra chinensis*. This study fills the research gap concerning flavor distribution across different fruit tissues. These findings also provide critical data to guide separate exploitation of *Schisandra chinensis* flesh and seeds.

## Data Availability

The original contributions presented in the study are included in the article/Supplementary material, further inquiries can be directed to the corresponding authors.

## Footnotes

1
https://pubchem.ncbi.nlm.nih.gov/

2
https://insilico-cyp.charite.de/VirtualTaste/
