# Supplementary material for: Taste and odor metabolomics of Schisandra chinensis flesh and seeds: integration of sensory evaluation, electronic tongue, LC-QTOF-MS/MS, VirtualTaste, and HS-GC-IMS
Source: Front Nutr. 2026 Aug 5;13:1881293. doi: 10.3389/fnut.2026.1881293 (PMC13485799; doi:10.3389/fnut.2026.1881293)
Supplement: Supplementary file 1 [file Table_1.DOCX]

Taste and odor metabolomics of Schisandra chinensis flesh and seeds: integration of sensory evaluation, electronic tongue, LC-QTOF-MS/MS, VirtualTaste, and HS-GC-IMS

Tian-Qi Zhang^1^, Lichao Wang^2^, Fang-Tong Liu^1^, Gui-Zhong Xin^3^, Hong-Juan Hou^1^, Jinxiu Liang^4^, Hui Zhang^1^, Tian-Min Wang^1^, De-Qiang Dou^1^, Xiaowei Zhang^5^, Da-Chuan Zhang^1^, Cheng Qian^6^, Long-Jiao Cong^1^, Ting-Guo Kang^1^, Gui-Rong Chen^1,*^, Yue-Hua Chen^1,*^, Hui-Peng Song^1,*^

^1^ Key Laboratory for Identification and Quality Evaluation of TCM of Liaoning Province, Liaoning University of Traditional Chinese Medicine, Dalian, China.

^2^ Department of Integration of Chinese and Western Medicine, School of Basic Medical Sciences, Peking University, Beijing, China.

^3^ State Key Laboratory of Natural Medicines, China Pharmaceutical University, Nanjing, China.

^4^ Institute of Genetics, International School of Medicine, Zhejiang University , Hangzhou, China.

^5^ Academy of Chinese Medical Sciences, Henan University of Chinese Medicine, Zhengzhou, China.

^6^ Department of Pathology, School of Basic Medical Sciences, Nanjing Medical University, Nanjing, China.

Corresponding authors:

**Hui-Peng Song**	E-mail: [songhuipeng15@163.com](mailto:songhuipeng15@163.com)

**Yue-Hua Chen**	E-mail: [chenyuehua527@126.com](mailto:chenyuehua527@126.com)

**Gui-Rong Chen**	E-mail: [cgr800404@sohu.com](mailto:cgr800404@sohu.com)

# Supplementary Figures and Tables

**Figure S1** Total ion current (TIC) chromatograms of (A) malic acid, (B) citric acid, (C) protocatechuic acid, (D) schisandrol A, (E) schisandrol B, (F) schisandrin A, (G) schisantherin A, (H) schisantherin B, (I) schisanhenol, and (J) schisandrin B

**Figure S2** Calibration curves for schisantherin A, schisandrin A, and schisandrin B

**Figure S3** Comparative chromatograms of the blank matrix, flesh, and seeds

**Table S1** Information of compounds in *Schisandra chinensis*

| No. | Identification | Formula | Molecular weight |
| --- | --- | --- | --- |
| 1; 2; 3; 4; 5; 6; 7; 8; 9; 10; 11; 12; 13; 14; 15; 16; 17; 18; 19; 20; 21; 22; 23; 24; 25; 26; 27; 28; 29; 30; 31; 32; 33; 34; 35; 36; 37; 38; 39; 40; 41; 42; 43; 44; 45; 46; 47; 48; 49; 50; 51; 52; 53; 54; 55; 56; 57; 58; 59; 60; 61; 62; 63; 64; 65; 66; 67; 68; 69; 70; 71; 72; 73; 74; 75; 76; 77; 78; 79; 80; 81; 82; 83; 84; 85; 86; 87; 88; 89; 90; 91; 92; 93; 94; 95; 96; 97; 98; 99; 100; 101; 102; 103; 104; 105; 106; 107; 108; 109; 110; 111 | Schisandrol A; Schisandrol B; Schisandrin A; Schisandrin B; Schisandrin C; Gomisin D; Gomisin J; Gomisin G; Gomisin H; Epigomisin O; Schisantherin A; Schisantherin D; Tigloylgomisin H; Tigloylgomisin P; Angeloylgomisin Q; Angeloylgomisin O; Benzoylisogomisin O; 3-Hydroxymethyl-2-furaldehyde; Myristic acid; β-Bisabolene; Mesodihydroguaiaretic acid; Chamigrenal; Nigranoic acid; Kadsuric acid; Gomisin M2; Gomisin S; Gomisin T; Gomisin K1; Gomisin L1; γ-Schisandrin; 6-O-Benzoylgomisin O; Deoxyschisandrin; Schisanhenol; Gomisin R; Gomisin K3; Angeloylgomisin R; Tigloylgomisin H; Angeloylgomisin P; Schisantherin B; Pre-schisanartanin A; Pre-schisanartanin B; Schicagenin A; Schicagenin B; Arisanlactone A; Arisanlactone B; Lancifodilactone D; Lancifodilactone F; Wuweizidilactone H; Wuweizidilactone I; Wuweizidilactone K; Wuweizidilactone L; Wuweizidilactone M; Wuweizidilactone N; Wuweizidilactone O; Micradilactone A; Micradilactone B; Schindilactone A; Schindilactone B; Schindilactone C; Schindilactone D; Schisanlactone C; D-Epigalbacin; Methyl acetoxyangolensate; 5,6-Epoxy-8,11,14-eicosatrienoic acid; Leukotriene A4; Leukotriene C4; Leukotriene E4; Mesobilirubinogen; Bilirubin; Sphingosine 1-phosphate; Sphinganine 1-phosphate; Linoleic acid; Alpha-Linolenic acid; Arachidonic acid; Docosahexaenoic acid; Oleic acid; L-Leucine; Indoleacrylic acid; Taurocholic acid; Cortexolone; 17-beta-estradiol-3-glucuronide; 6-Keto-prostaglandin F1a; Oxoglutaric acid; Citric acid; Uric acid; D-Fructose; Inositol 1,3,4,5-tetraphosphate; 11-cis-Retinol; Retinoyl beta-glucuronide; Retinyl ester; Clupanodonic acid; Hyperoside; Rutin; Protocatechuic acid; Thymoquinol 5-O-glucoside; Schisandenoid A; Isoquercitrin; Tetrahydroxyhexuronic acid glycoside; Schizanlactone; Kadsuphilactone A; Ursolic acid; Oleanolic acid; Gallic acid; Palmitic acid; Stearic acid; Lauric acid; Capric acid; Myristoleic acid; Palmitoleic acid; Vaccenic acid; Elaidic acid | C₂₄H₃₂O₇; C₂₃H₂₈O₇; C₂₄H₃₂O₆; C₂₃H₂₈O₆; C₂₂H₂₄O₆; C₂₈H₃₄O₁₀; C₂₂H₂₈O₆; C₃₀H₃₂O₉; C₂₃H₃₀O₇; C₂₃H₂₈O₇; C₃₀H₃₂O₉; C₂₉H₂₈O₉; C₂₈H₃₆O₈; C₂₈H₃₄O₉; C₂₉H₃₈O₉; C₂₈H₃₄O₈; C₃₀H₃₂O₈; C₆H₆O₃; C₁₄H₂₈O₂; C₁₅H₂₂; C₁₈H₂₂O₄; C₁₅H₂₂O; C₃₀H₄₆O₄; C₃₀H₄₆O₃; C₂₂H₂₆O₆; C₂₃H₃₀O₇; C₂₃H₃₀O₇; C₂₃H₃₀O₆; C₂₂H₂₆O₆; C₂₃H₂₈O₆; C₃₀H₃₂O₈; C₂₄H₃₂O₆; C₂₃H₃₀O₆; C₂₂H₂₄O₇; C₂₃H₃₀O₆; C₂₈H₃₄O₈; C₂₈H₃₆O₈; C₂₈H₃₄O₉; C₂₈H₃₄O₉; C₂₉H₃₆O₁₁; C₂₉H₃₆O₁₁; C₂₉H₃₆O₁₂; C₂₉H₃₆O₁₁; C₂₉H₃₆O₁₂; C₂₉H₃₆O₁₂; C₂₉H₃₄O₉; C₂₉H₃₄O₁₀; C₂₈H₃₆O₁₀; C₃₃H₄₂O₁₁; C₃₁H₄₂O₁₀; C₂₉H₃₆O₉; C₂₉H₃₆O₉; C₂₈H₃₆O₉; C₂₈H₃₄O₉; C₂₉H₃₆O₁₂; C₂₉H₃₆O₁₁; C₂₉H₃₄O₁₁; C₂₉H₃₄O₁₁; C₂₉H₃₆O₁₁; C₂₉H₃₄O₁₂; C₃₀H₄₀O₅; C₂₈H₃₄O₇; C₂₉H₃₆O₉; C₂₀H₃₂O₃; C₂₀H₃₀O₃; C₃₀H₄₇N₃O₉S; C₂₃H₃₇NO₅S; C₃₃H₄₄N₄O₆; C₃₃H₃₆N₄O₆; C₁₈H₃₈NO₅P; C₁₈H₄₀NO₅P; C₁₈H₃₂O₂; C₁₈H₃₀O₂; C₂₀H₃₂O₂; C₂₂H₃₂O₂; C₁₈H₃₄O₂; C₆H₁₃NO₂; C₁₁H₉NO₂; C₂₆H₄₅NO₇S; C₂₁H₃₀O₄; C₂₄H₃₂O₈; C₂₀H₃₄O₆; C₅H₆O₅; C₆H₈O₇; C₅H₄N₄O₃; C₆H₁₂O₆; C₆H₁₆O₁₈P₄; C₂₀H₃₀O; C₂₆H₃₆O₈; C₂₀H₃₀O₂; C₂₂H₃₄O₂; C₂₁H₂₀O₁₂; C₂₇H₃₀O₁₆; C₇H₆O₄; C₁₆H₂₄O₇; C₂₂H₃₄O₁₁; C₂₁H₂₀O₁₂; C₁₈H₂₄O₁₈; C₃₀H₄₀O₅; C₃₅H₄₄O₁₂; C₃₀H₄₈O₃; C₃₀H₄₈O₃; C₇H₆O₅; C₁₆H₃₂O₂; C₁₈H₃₆O₂; C₁₂H₂₄O₂; C₁₀H₂₀O₂; C₁₄H₂₆O₂; C₁₆H₃₀O₂; C₁₈H₃₄O₂; C₁₈H₃₄O₂ | 432.51; 416.46; 416.51; 400.46; 384.42; 530.56; 388.45; 536.57; 418.48; 416.46; 536.57; 520.53; 500.58; 514.56; 530.6; 498.56; 512.57; 126.11; 228.37; 202.33; 302.36; 218.33; 470.68; 454.68; 386.44; 418.48; 418.48; 402.48; 386.44; 400.46; 512.57; 416.51; 402.48; 400.42; 402.48; 498.56; 500.58; 514.56; 514.56; 560.59; 560.59; 576.59; 560.59; 576.59; 576.59; 526.57; 542.57; 532.56; 614.67; 574.64; 540.6; 540.6; 516.56; 514.54; 576.59; 560.59; 558.57; 558.57; 560.59; 574.57; 484.63; 486.56; 540.6; 320.47; 318.45; 625.78; 439.61; 592.73; 584.66; 379.47; 381.48; 280.44; 278.43; 304.47; 328.49; 282.46; 131.17; 187.19; 515.7; 346.46; 448.5; 370.48; 146.1; 192.12; 168.11; 180.16; 499.99; 286.45; 476.56; 302.45; 334.5; 464.38; 610.52; 154.12; 328.36; 474.5; 464.38; 492.37; 484.63; 656.71; 456.7; 456.7; 170.12; 256.42; 284.48; 200.32; 172.26; 226.36; 254.41; 282.46; 282.46 |

**Table S2** Inter-day precision of peak areas for schisantherin A, schisandrin A, and schisandrin B

| Number of injections | 1 | 2 | 3 | 4 | 5 | 6 | RSD(%) |
| --- | --- | --- | --- | --- | --- | --- | --- |
| Schisantherin A | 261.849 | 261.635 | 263.972 | 261.414 | 262.871 | 261.733 | 0.38 |
| Schisandrin A | 775.359 | 777.181 | 793.149 | 783.468 | 783.871 | 791.033 | 0.91 |
| Schisandrin B | 785.075 | 787.325 | 798.404 | 789.645 | 789.47 | 801.055 | 0.81 |

**Table S3** Intra-day precision of peak areas for schisantherin A, schisandrin A, and schisandrin B

| Number of injections | 1 | 2 | 3 | 4 | 5 | 6 | RSD(%) |
| --- | --- | --- | --- | --- | --- | --- | --- |
| Schisantherin A | 261.434 | 266.451 | 264.28 | 254.287 | 261.836 | 256.613 | 1.76 |
| Schisandrin A | 771.194 | 774.364 | 774.824 | 779.173 | 783.033 | 783.468 | 0.64 |
| Schisandrin B | 787.521 | 782.309 | 790.753 | 781.836 | 793.443 | 789.311 | 0.59 |

**Table S4** Matrix effect of the seeds

| Sample | Measured content of stock solution (mg/mL) | Stock content calculated by 2-fold dilution (mg/mL) | Stock content calculated by 3-fold dilution (mg/mL) | Stock content calculated by 4-fold dilution (mg/mL) | Average content (mg/mL) | RSD (%) |
| --- | --- | --- | --- | --- | --- | --- |
| Schisantherin A | 0.0420 | 0.0424 | 0.0454 | 0.0442 | 0.0435 | 3.72 |
| Schisandrin A | 0.0493 | 0.0504 | 0.0509 | 0.0489 | 0.0499 | 1.88 |
| Schisandrin B | 0.0523 | 0.0543 | 0.0518 | 0.0514 | 0.0524 | 2.47 |

**Table S5** Matrix effect of the flesh

| Sample | Measured content of stock solution (mg/mL) | Stock content calculated by 2-fold dilution (mg/mL) | Stock content calculated by 3-fold dilution (mg/mL) | Stock content calculated by 4-fold dilution (mg/mL) | Average content (mg/mL) | RSD (%) |
| --- | --- | --- | --- | --- | --- | --- |
| Schisantherin A | / | / | / | / | / | / |
| Schisandrin A | 0.0047 | 0.0049 | 0.0050 | 0.0049 | 0.0049 | 2.19 |
| Schisandrin B | 0.0065 | 0.0064 | 0.0069 | 0.0066 | 0.0066 | 3.30 |
